# Evaluation of the Antiproliferative Properties of CpRu Complexes Containing N-Methylated Triazaphosphaadamantane Derivatives

**DOI:** 10.1155/2023/6669394

**Published:** 2023-09-28

**Authors:** Andres Alguacil, Franco Scalambra, Antonio Romerosa, Andreia Bento-Oliveira, Fernanda Marques, Ines Maximiano, Rodrigo F. M. de Almeida, Ana Isabel Tomaz, Andreia Valente

**Affiliations:** ^1^Área de Química Inorgánica–CIESOL, Universidad de Almería, Almería 04120, Spain; ^2^Centro de Química Estrutural, Institute of Molecular Sciences, Departamento de Química e Bioquímica, Faculdade de Ciências, Universidade de Lisboa, Campo Grande 1749-016, Lisboa, Portugal; ^3^Centro de Ciências e Tecnologias Nucleares e Departamento de Engenharia e Ciências Nculeares, Instituto Superior Técnico (C2TN/IST), Universidade de Lisboa, Estrada Nacional 10, Bobadela LRS 2695-066, Portugal

## Abstract

Piano-stool-{CpRu} complexes containing 1,3,5-triaza-7-phosphaadamantane (PTA), *N*-methyl-1,3,5-triaza-7-phosphaadamantane (mPTA), and 3,7-dimethyl-1,3,7-triaza-5-phosphabyciclo[3.3.1]nonane (dmoPTA) were evaluated as drugs against breast cancer. The evaluated compounds include two new examples of this family, the complexes [RuCp(DMSO-*κ*S)(HdmoPTA)(PPh_3_)](CF_3_SO_3_)_2_ (**8**) and [RuCp(PPh_3_)_2_-*µ*-dmoPTA-1*κP*-2*κ*^2^*N*,*N*′-PdCl_2_](CF_3_SO_3_) (**11**), which have been synthesized and characterized by NMR, IR, and single-crystal X-ray diffraction. The cytotoxic activity of compounds was evaluated against MDA-MB-231 breast cancer cells, and the three most active complexes were further tested against the hormone-dependent MCF-7 breast cancer cell line. Their cell death mechanism and ruthenium uptake were also evaluated, as well as their binding ability to human serum albumin.

## 1. Introduction

The development of new drugs for the treatment of cancer is certainly a topic of preeminent importance. Since the discovery of the anticancer activity of *cis*-[PtCl_2_(NH_3_)_2_] (cisplatin) by Rosenberg et al. in the 1960s [1, 2], large research efforts have been focused on the development of new platinum-based drugs, some of which are currently used in numerous chemotherapeutic treatments [3–5]. However, their side effects, lack of selectivity, and resistance-related issues pushed the scientific community to explore complexes with similar geometry and chemical properties but containing other metals such as Pd(II) [6–8]. This strategy has led to the recent regulatory approval in Europe of the Pd-porphyrin complex padeliporfin (WST11) for photodynamic therapy treatments [9].

**Figure 1:**
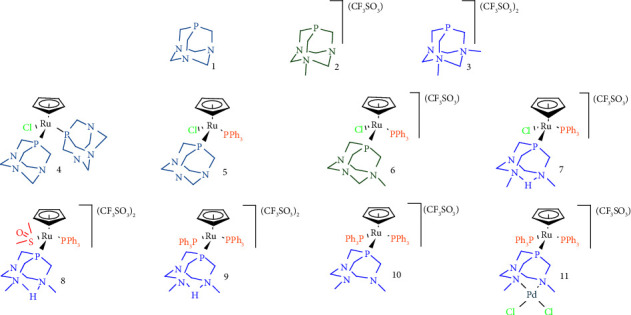
PTA-type ligands (**1**–**3**) and their RuCp complexes (**4**–**11**) were studied in this work and screened for their anticancer activity in the breast model.

Research efforts targeted to obtain alternatives to Pt(II) compounds have led to the development of new complexes containing metals that adopt different coordination geometries concerning the strictly square planar of Pt(II) and Pd(II). Among the transition metals that meet this requirement, ruthenium is one of the most promising [10–12]. Several ruthenium-based compounds have shown outstanding encouraging results such as Ru(III) complexes KP1019 (replaced by NKP1339/IT-139) and NAMI-A, which entered phases I and II of clinical trials, respectively [12]. Other examples are the Ru(II) complex TLD1433, which was evaluated in phase II [13, 14], and the compounds of the family of RAPTAs, which were tested in preclinical trials [15–17]. This latter group of complexes exhibits a Ru-(*η*^6^-arene) scaffold and 1,3,5-triaza-7-phosphaadamantane (PTA, **1**) together with two chlorides (Figure 1) [18]. The combination of ligands and their arrangement around the metal provides a stable platform with adequate hydrosolubility, stabilizing the Ru(II) oxidation state [19].

To date, and inspired by the RAPTA family, a discrete number of antiproliferative ruthenium compounds bearing PTA and derivatives have been synthesized [20–22]. In this line, complexes containing 3,7-dimethyl-1,3,7-triaza-5-phosphabicyclo[3.3.1]nonane (dmoPTA), which is a hydrolysis product of N,N′-dimethyl-1,3,5-triaza-7-phosphaadamantane (dmPTA) (**3**, Figure 1), display a very good antiproliferative profile [23], surpassing the activity of cisplatin up to 200 times in some cases [24]. Some of the most active examples of this family of compounds are heterometallic complexes in which the dmoPTA ligand is binding ruthenium by the phosphorus atom and chelates harder metals through methylated nitrogens [24–29], and have been studied along with other complexes presented in this work together with the free ligands by ^15^N NMR. [30].

The reasons that determine the significant cytotoxic activity of heterometallic complexes containing dmoPTA are not still envisaged. As a part of our efforts to shed light on this topic, some significantly active previously published complexes containing PTA and PTA derivatives (Figure 1) were compared with the two new members of this family: monometallic [RuCp(DMSO-*κ*S)(HdmoPTA)(PPh_3_)](CF_3_SO_3_)_2_ (**8**) and bimetallic [RuCp(PPh_3_)_2_-*µ*-dmoPTA-1*κP*-2*κ*^2^*N,N*′-PdCl_2_](CF_3_SO_3_) (**11**). Complexes **1**–**11** were evaluated against the MDA-MB-231 breast cancer cell line, and the most active compounds (**9**–**11**) were further tested against the MCF-7 breast cancer cell line. In addition, their cell death mechanism and cell distribution (Ru quantification) were also determined. To gain insight into the possible role of human serum albumin (HSA) in their bloodstream distribution, their interaction with HSA was also quantified using steady-state and time-resolved fluorescence.

## 2. Experimental

### 2.1. Materials and Methods

All chemicals were reagent grade and, unless otherwise stated, were used as received by commercial suppliers. In the synthesis of the new compounds, all solvents used were degassed and distilled according to standard procedures. All reactions and manipulations were routinely performed under a dry nitrogen atmosphere using standard Schlenk-tube techniques. Hydrosoluble phosphines PTA (**1**) [18], mPTA(CF_3_SO_3_) (**2**) [31], and dmPTA(CF_3_SO_3_)_2_ (**3**) [23] and complexes [RuClCp(PTA)_2_] (**4**) [32], [RuClCp(PPh_3_)(PTA)] (**5**) [31], [RuClCp(PPh_3_)(mPTA)](CF_3_SO_3_) (**6**) [31], [RuClCp(HdmoPTA)(PPh_3_)](CF_3_SO_3_) (**7**) [23], [RuCp(HdmoPTA)(PPh_3_)_2_](CF_3_SO_3_)_2_ (**9**) [27], and [RuCp(dmoPTA)(PPh_3_)_2_](CF_3_SO_3_) (**10**) [24] were prepared as described in the literature. ^1^H, ^13^C{^1^H}, and ^31^P{^1^H} NMR spectra were recorded on a Bruker DRX500 spectrometer operating at 500.13 MHz, 150.77 MHz, and 202.46 MHz, respectively. Peak positions are relative to tetramethylsilane and were calibrated against the residual solvent resonance (^1^H) or the deuterated solvent multiplet (^13^C). Chemical shifts for ^31^P{^1^H} NMR were measured relative to external 85% H_3_PO_4_ with downfield values taken as positive. Elemental analysis (C, H, N, S) was performed on a Fisons Instruments EA 1108 elemental analyser. Infrared spectra have been carried out on a Bruker ECO-ATR ALPHA spectrometer, and the intensity of the bands has been indicated as strong (s), medium (m), and weak (w). The electronic absorption spectra were acquired on Jasco UV-1603 or a Jasco V-560 spectrophotometer (JASCO, Hiroshima, Japan) at room temperature with 1 cm optical path quartz Hellma cuvettes. The solubility of the synthesized complexes was determined by UV-vis spectrophotometry techniques. Fluorescence measurements were performed on a Fluorolog 3.22 spectrofluorometer from Horiba Jobin Yvon (Villeneuve D'ascq, France) at (25.0 ± 0.1)°C.

### 2.2. Synthetic Procedures

#### 2.2.1. Synthesis of [RuCp(DMSO-*κ*S)(HdmoPTA)(PPh_3_)](CF_3_SO_3_)_2_ (**8**)

32.6 mg of AgCF_3_SO_3_ (0.127 mmol) was added into a solution of 50 mg of [RuClCp(HdmoPTA)(PPh_3_)](CF_3_SO_3_) (**7**) (0.0635 mmol) in 0.5 mL of DMSO. A white AgCl precipitate was formed, and the mixture was stirred for 24 h, filtered with celite, and washed with 1 mL of EtOH. 5 mL of Et_2_O was added to the filtered solution, and a whitish precipitate appeared. It was filtered and washed with Et_2_O (3 × 3 mL) and left to dry under vacuum. Yield: 53.5 mg, 86%. Solubility: S_25°C,acetone_ 40.8 mg/cm^3^, S_25°C,MeOH_ 19.2 mg/cm^3^, and S_25°C,DMSO_ 76.4 mg/cm^3^. C_34_H_43_F_6_N_3_O_7_P_2_RuS_3_ (979.07 g·mol^−1^). Calcd: C 41.67; H 4.26; N 4.29; S 9.79. Found: C 41.27; H 4.52; N 4.47; S 9.51. IR: 1438 (w), 1252 (s), 1226 (m), 1151 (m), 1097 (w), 1033 (s), 997 (m), 934 (m), 756 (m), 704 (m), 698 (m), and 641 (s). ^1^H NMR (500.13 MHz, acetone-d_6_, 25°C) *δ* (ppm): 2.50 + 2.54 (*d* + *d*, ^2^*J*_HH_ = 1.46 Hz, ^2^*J*_HH_ = 1.44 Hz, 3H + 3H, CH_3_NCH_2_P_HdmoPTA_), 3.01 + 3.67 (*s* + *s*, 3H + 3H, (CH_3_)_2_SO), 3.42–3.66 + 4.30 (*m* + *m*, 6H, NCH_2_P_HdmoPTA_), 4.02 + 4.51 (*d* + *m*, ^2^*J*_HH_ = 11.61 Hz, 4H, NCH_2_N_HdmoPTA_), 5.44 (s, 5H, Cp), and 7.56–7.67 (m, 15H, PPh_3_). ^13^C{^1^H} NMR (125.76 MHz, acetone-d_6_, 25°C) *δ* (ppm): 42.31 + 42.45 (*d* + *d*, ^3^*J*_PC_ = 5.24 Hz, ^3^*J*_PC_ = 4.93 Hz, CH_3_NCH_2_P_HdmoPTA_), 54.23 + 58.98 (*s* + *s*, (CH_3_)_2_SO), 48.16 + 48.34 + 55.68 + 55.85 + 56.24 + 56.40 (*m* + *m* + *m* + *m*, NCH_2_P_HdmoPTA_), 74.97 + 75.05 (*d* + *d*, ^3^*J*_PC_ = 3.74 Hz, ^3^*J*_PC_ = 3.20 Hz, NCH_2_N_HdmoPTA_), 85.72 (s, Cp), and 129.16–133.90 (m, PPh_3_). ^31^P{^1^H} NMR (202.46 MHz, acetone-d_6_, 25°C) *δ* (ppm): −5.89 (d, ^2^*J*_PP_ = 37.64 Hz, HdmoPTA), and 41.48 (d, ^2^*J*_PP_ = 37.45 Hz, PPh_3_).

#### 2.2.2. Synthesis of [RuCp(PPh_3_)_2_-*µ*-dmoPTA-1*κ*P-2*κ*^2^N,N′-PdCl_2_](CF_3_SO_3_) (**11**)

(NBu_4_)_2_[PdCl_4_] (68 mg, 0.098 mmol) was added into a solution of [RuCp(PPh_3_)_2_(dmoPTA)](CF_3_SO_3_) (**10**) (100 mg, 0.098 mmol) in dry MeOH (20 mL). The mixture was stirred at room temperature for 30 minutes, and the solvent was removed. The pale brown residue was dissolved in CHCl_3_ (10 mL), and it was filtered by gravity. The filtered dissolution was concentrated under reduced pressure, and Et_2_O was added drop to drop until a pale brown precipitate was observed. The resulting precipitate was suction filtered, washed with Et_2_O (3 × 20 mL), and dried under vacuum. Yield: 62.9 mg, 54%. Solubility: S_25°C,CHCl3_ 4.8 mg/cm^3^, S_25°C,DMF_ 17 mg/cm^3^, and S_25°C,DMSO_ 25.2 mg/cm^3^. C_49_H_51_Cl_2_F_3_N_3_O_3_P_3_PdRuS (1189,03 g·mol^−1^). Calcd: C 49.45; H 4.32; N 3.53; S 2.69. Found: C 49.64; H 4.47; N 3.39; S 2.48. IR: 2958 (w); 1433 (m); 1275 (s), 1158 (s); 826 (s). ^1^H NMR (600.13 MHz, CDCl_3_, 25°C) *δ* (ppm): 2.45 (s, 6H, N*CH*_*3*_), 3.10 + 4.01 (*m* + *m*, 4H, PCH_2_NCH_3_), 3.45 (m, 2H, PCH_2_N), 4.24 (m, 4H, NCH_2_N), 5.16 (s, 5H, Cp), and 6.89–7.60 ppm (m, 30 H, aromatics, PPh_3_). ^13^C{^1^H} NMR (150.90 MHz, CDCl_3_, 25°C) *δ* (ppm): 53.04 (m, NCH_3_), 60.27 (m, PCH_2_N), 61.77 (m, PCH_2_NCH3), 78.25 (m, NCH_2_N), 86.50 (s, Cp), and 128.45–136.51 (PPh_3_). ^31^P{^1^H} NMR (242.94 MHz, CDCl_3_, 20°C) *δ* (ppm): −10.48 (m, dmoPTA-PdCl_2_), and 39.51 (d, ^2^*J*_PP_ = 38.92 Hz, PPh_3_).

### 2.3. Single-Crystal X-Ray Diffraction

Single crystals of **8** and **11** were obtained by evaporation of acetone and CHCl_3_ solutions, respectively. Single-crystal X-ray diffraction measurements were performed with a Bruker APEX-II CCD diffractometer using MoK*α* radiation. Data were integrated (SAINT, Bruker) and scaled (SADABS, Bruker), and finally, the structure was solved with SHELXT using intrinsic phasing and refined with SHELXL by least squares [33, 34]. Solution and refinement procedures were accomplished by using Olex2 software [35]. The crystal structures of **8** and **11** have been deposited at CSD with CCDC numbers 2263437 (**8**) and 2261422 (**11**). The crystallographic and experimental parameters are summarized in Table S1. Distances and angles are summarized in Tables S2–S6.

### 2.4. Biological Evaluation

#### 2.4.1. Stability Studies

The assessment of the biological properties of the complexes requires their dissolution in a cell culture medium which is mainly constituted by water. When the complex is not hydrosoluble enough, the used protocol contemplates preliminary dissolution in DMSO before the addition to the culture medium [36]. Thus, it is important to determine the stability and behaviour of the complexes in both DMSO and water/DMSO mixtures to check whether different species have been formed when dissolved. Dissolutions in DMSO-d_6_ and DMSO-d_6_/D_2_O of the new complexes **8** and **11** were monitored by ^31^P{^1^H} NMR over time at room temperature and 37°C. The stability studies of the rest of the complexes (1‐7, 9, 10) were published previously. All experiments were performed by a similar procedure: the complex (0.01 g) was introduced into a 5 mm NMR tube and dissolved in 0.5 mL of degassed solvent (DMSO-d_6_ and a 1 : 1 mixture of DMSO-d_6_/D_2_O). The solution was left at room temperature and monitored by ^31^P{^1^H} NMR first every 15 minutes, and later in longer periods. Stability was also monitored at incubation cell-based assay temperature (37°C).

#### 2.4.2. Cell Lines and Culture Conditions

The MDA-MB-231 and MCF-7 human breast cancer cells were purchased from ATCC. The MDA-MB-231 and MCF-7 cells were grown in Dulbecco's modified Eagles' medium (DMEM high glucose) (Capricorn Scientific) at 37°C in 5% CO_2_ supplemented with 10% fetal bovine serum (Capricorn Scientific). HDF cells were purchased from Merck and were grown in the fibroblast growth medium. All cells were adherent in monolayers and, upon confluence, were washed with phosphate buffer saline (PBS) and harvested by digestion with trypsin-EDTA 0.05% (v/v). The cells were suspended and transferred into new, sterile, culture flasks for maintenance or seeded in sterile test microplates for different assays. All cells were manipulated under aseptic conditions in a flow chamber.

#### 2.4.3. Compound Cytotoxicity Evaluated Using the MTT Assay

The cells were adherent in monolayers and, upon confluence, were harvested by digestion with trypsin. The cytotoxicity of the complexes against the tumour cells was assessed using the colorimetric assay MTT (3-(4,5-2-yl)-2,5-ditetrazolium bromide), which evaluates the metabolic activity of viable cells. This assay measures the conversion of yellow tetrazolium into purple formazan by an active mitochondrial dehydrogenase in living cells. For this purpose, the cells (10–20 × 10^3^ in 200 *μ*L of the medium) were seeded into 96-well plates and incubated in a 5% CO_2_ incubator at 37°C. The cells were settled for 24 h followed by the addition of a dilution series of the complexes in a medium (200 *μ*L). The complexes were first solubilized in 100% DMSO, given a 10 mM stock solution, and then in a medium within the concentration range of 0.1–100 *µ*M. DMSO did not exceed 1% even for the higher concentration used and was without cytotoxic effects. After 24 h of incubation, the treatment solutions were removed by aspiration, and MTT solution (200 *μ*L, 0.5 mg·mL^−1^ in PBS) was added to each well. After 3 h at 37°C/5% CO_2_, the solution was removed, and the purple formazan crystals formed inside the cells were dissolved in DMSO (200 *μ*L) by thorough shaking. The cellular viability was evaluated by measuring the absorbance at 570 nm by using a microplate spectrophotometer. The IC_50_ values were obtained by dose-response curves using the GraphPad Prism software (vs. 5).

#### 2.4.4. Cell Death Measurement Using Flow Cytometry: The Annexin V/PI Assay

After 24 h treatment with compounds **9**, **10**, and **11**, both suspended and attached cells were collected and washed with PBS. The cells were resuspended in 200 *μ*L of 1x binding buffer and were incubated with 5 *μ*L of FITC annexin V (BD Biosciences, San Jose, CA, USA) and 10 *μ*L of PI (50 mg·mL^−1^) for 20 min in the dark. The samples were analysed by using fluorescence-activated cell sorting (FACS) using Beckman Coulter EPICS XL-MCL. All data were analysed using the FlowJo software (version 10, Tree Star Inc.).

#### 2.4.5. Complex Uptake and Distribution by ICP-MS

For the cellular uptake experiments, MDA-MB-231 cells (approx. 5·10^6^ cells/5 mL medium) were exposed to the complexes at their respective IC_50_ concentrations and in two conditions (6 h and 24 h of incubation time) and then washed with ice-cold PBS and centrifuged to obtain a cellular pellet [37]. The cytosol, membrane/particulate, cytoskeletal, and nuclear fractions were extracted using a FractionPREP™ cell fractionation system (BioVision, USA) and performed according to the manufacturer's protocol. The Ru (^101^Ru) and Pd (^106^Pd) contents in different fractions were measured by using Thermo X-Series Quadrupole ICP-MS (Thermo Scientific) after the digestion of the samples. Briefly, the samples were digested with ultrapure HNO_3_ (65%), H_2_O_2_, and H_3_PO_4_ in a closed pressurized microwave digestion unit (Mars5, CEM) with medium-pressure HP500 vessels and then diluted in ultrapure water to obtain 2.0% (v/v) nitric acid. The instrument was tuned using a multielement ICP-MS 71C standard solution (Inorganic Venture). Indium (115In) at 10*μ*ML^−1^ was used as the internal standard.

#### 2.4.6. Interaction with Human Serum Albumin

The stock solutions of HSA were freshly prepared for each experiment by gently dissolving protein in PBS pH 7.4 (tablets from Fisher) for 1 hour to allow protein to completely hydrate. The concentration of the protein in the stock solutions was determined by spectrophotometry using the molar absorption coefficient at 280 nm *ε*_280_(HSA) = 36,850 M^−1^·cm^−1^ [38].

In each essay, all spectroscopic measurements were carried out on individually prepared samples to ensure the same preincubation time at (37.0 ± 0.1)°C, the same % of DMSO in the final samples, and the same exposure to excitation light and to avoid the need for dilution corrections. Moreover, for a direct titration protocol, a highly concentrated stock solution of the compounds in the buffer would be required, which would raise solubility issues. Dimethylsulfoxide (DMSO, from Fisher) was used to prepare concentrated stock solutions of each complex, followed by appropriate dilution to obtain the desired concentration. The DMSO content was kept at 2% (v/v) in PBS pH 7.4 in all samples. Dilutions were carried out immediately before sample preparation.

Fluorescence measurements were performed at 25.0 ± 0.1°C. The final protein concentration in the individually prepared samples (3.72 to 5 *μ*M) was kept constant in each assay, and the complex concentration was varied to obtain desired HSA: Ru-complex molar ratios ranging from 1 : 0.25 to 1 : 10. A control sample containing the same amount of protein and final DMSO content and with no complex was also prepared for each experiment. Appropriate blank samples with no protein but with the same complex concentrations were prepared as well for background correction.

The steady-state fluorescence intensity was measured with excitation at 295 nm, and emission spectra were collected with bandwidths of 4 nm for both excitation and emission. For Stern–Volmer plots, the fluorescence intensity with emission at 340 nm was selected because it is near the maximum emission wavelength (so the sensitivity is close to maximum), but it is further to red in relation to the water Raman scattering peak [39]. These values were corrected for the absorption and emission inner filter effects [40] using the absorbance recorded for each sample at the excitation and emission wavelengths used for the steady-state quenching analysis. For time-resolved fluorescence measurements, the single-photon-counting technique was used with nanoLED N-280 (Horiba Jobin Yvon) as the pulsed excitation source (280 nm) and with emission collected at 350 nm (13 nm emission bandwidth), eliminating the contribution of the emission by tyrosine residues.

The experimental fluorescence intensity decays of HSA in buffer solution can be described by a sum of exponentials:

(1) $$\begin{array}{c} \left.I\left(t\right)=\overset{n}{\underset{i=1}{\sum }}p_{i}\exp\left(-\frac{t}{\tau_{i}}\right.\right)\text{,} \end{array}$$

where *p*_i_ and *τ*_i_ are the pre-exponential factors and lifetime of component *i*, respectively. The amplitude or normalized pre-exponential of each lifetime component *α*_*i*_ is *p*_*i*_/Σ_*i*_*p*_*i*_ with Σ_*i*_*α*_*i*_ = 1. Fluorescence decays were analysed by an iterative deconvolution method using the *TRFA Data Processor software* (version 1.4; Minsk, Belarus), and the instrument response function was obtained using scattering by a colloidal suspension of silica (Ludox®, Sigma-Aldrich) diluted in water. Criteria for judging the quality of the fit were reduced *χ*^2^ close to 1 and a random distribution of weighted residuals and residual autocorrelation.

To evaluate changes in the quantum yield by processes affecting the fluorescence lifetimes of HSA, the *amplitude-weighted mean fluorescence lifetime* ($\bar{\tau }$) was calculated using the following equation:

(2) $$\begin{array}{c} \bar{\tau }=\overset{n}{\underset{i=1}{\sum }}\alpha_{i}\tau_{i}\text{.} \end{array}$$

## 3. Results and Discussion

### 3.1. Characterization of Complexes **8** and **11**

**Scheme 1:**
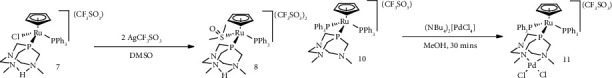
Synthesis of **8** (a) and **11** (b).

The complex [RuCp(DMSO-*κ*S)(HdmoPTA)(PPh_3_)](CF_3_SO_3_)_2_ (**8**) was synthesized by addition of AgCF_3_SO_3_ into a solution of **7** in DMSO (Scheme 1(a)), while the complex [RuCp(PPh_3_)_2_-*µ*-dmoPTA-1*κP*-2*κ*^2^*N,N*′-PdCl_2_](CF_3_SO_3_) (**11**) was obtained (Scheme 1(b)) by reaction in methanol of (NBu_4_)_2_[PdCl_4_] with [RuCp(PPh_3_)_2_(dmoPTA)](CF_3_SO_3_) (**10**) [24]. These complexes were obtained as whitish and pale brown solids, respectively, in moderate to good yield, and were characterized in the solid state (FTIR, XRD, and elemental analysis) and solution (^1^H, ^31^P{^1^H}- NMR, ^13^C{^1^H} NMR, and 2D NMR)–see supporting information for details (Figures S1–S6 for **8** and Figures S7–S12 for **11**).

At 25°C, complex **8** is soluble in MeOH (19.2 mg/cm^3^), acetone (40.8 mg/cm^3^), and DMSO (76.4 mg/cm^3^), while **11** is soluble in CHCl_3_ (4.8 mg/cm^3^), DMF (17 mg/cm^3^), and DMSO (25.2 mg/cm^3^). The ^1^H NMR resonances were assigned with the help of ^1^H-^1^H COSY and ^1^H-^13^C HSQC experiments (**8**: Figures S5-S6; **11**: Figures S11-S12), which are in agreement with the crystal structure of the complexes and display similar chemical shifts than those found for similar compounds [24, 28, 41]. It is interesting to point out that the ^1^H NMR spectrum of **8** shows the distinctive signals of *κ*S-coordinated DMSO (*δ* (ppm, acetone-d_6_) = 3.01 + 3.67, *s* + *s*), while no evidence for the *κO*-DMSO coordination was observed [26–28]. The ^31^P{^1^H} NMR spectra of **8** and **11** display the signal of PPh_3_ as a doublet (**8** : 41.48 ppm (^2^*J*_PP_ = 37.45 Hz), acetone-d_6_; **11** : 39.51 ppm (^2^*J*_PP_ = 38.92 Hz), CDCl_3_), while that of the dmoPTA ligand appears as a doublet in the spectra of **8** (−5.89 ppm, acetone-d_6_) and a triplet for **11** (−10.48 ppm, CDCl_3_), agreeing to those observed in analogue complexes and with their crystal structure [24, 28, 41]. CF_3_SO_3_^−^ counterions in both complexes were confirmed by ^13^C{^1^H} NMR and IR (**8**: *υ*_SO3_ = 1252 cm^−1^; **11**: *υ*_SO3_ = 1275 cm^−1^).

**Figure 2:**
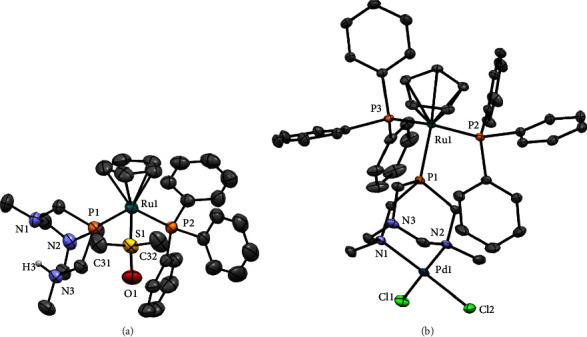
ORTEP view (50% probability) with atom numbering of the key atoms of the complex unit in the crystal structures of **8** (a) and **11** (b). Relevant bond lengths, plane, and torsion angles are given in Tables S2–S6 in SI. Anions, solvent molecules, and hydrogen atoms connected to the carbon atoms were omitted for clarity.

The asymmetric unit of **8** is constituted by the cationic complex [RuCp(DMSO-*κS*)(HdmoPTA)(PPh_3_)]^2+^, two triflate counterions, and one molecule of acetone, while the asymmetric unit of **11** contains one bimetallic cationic complex [RuCp(PPh_3_)_2_-*µ*-dmoPTA-1*κP*-2*κ*^2^*N,N*′-PdCl_2_]^+^ (Figure 2), one disordered triflate, and four molecules of CHCl_3_. The coordination sphere around the Ru atom in both complexes shows the expected distorted pseudooctahedral geometry. The Ru-Cp_centroid_ distance (**8** : 1.887·Å; **11** : 1.885·Å) is similar to that found in similar complexes [23–28, 41]. The angle between the Cp plane and the P1-Ru1-P2 plane is 51.24° for **8** and 49.14° for **11**, which are slightly smaller than those found in [RuCp(PPh_3_)_2_-*µ*-dmoPTA-1*κP*-2*κ*^2^*N,N*′-ZnCl_2_](CF_3_SO_3_) (51.20°), [RuCp(PPh_3_)_2_-*µ*-dmoPTA-1*κP*-2*κ*^2^*N,N*′-CoCl_2_](CF_3_SO_3_) (52.85°), and [RuCp(DMSO-*κ*S)(PPh_3_)(PTA)](CF_3_SO_3_) (52.82°) [24, 28, 41].

Bond lengths between ruthenium and P_dmoPTA_ in **8** and **11** are almost identical (**8**: Ru1-P1 = 2.3107(8) Å; **11**: Ru1-P1 = 2.3077(5) Å) and slightly shorter than those in similar previously reported complexes (**9** : 2.321(1) Å; **10** : 2.319(1) Å; [RuCp(PPh_3_)_2_-*µ*-dmoPTA-1*κ*P-2*κ*^2^*N,N*′-ZnCl_2_](CF_3_SO_3_): 2.327(1) Å) [24, 27]. The Ru-P_PPh3_ bond length for **8** was found to be a bit longer (2.3635(9) Å) than that in [RuCp(DMSO-*κS*)(PPh_3_)(PTA)](CF_3_SO_3_) (2.333(1) Å) [41]. Bimetallic complex **11** displays somewhat larger Ru-P_PPh3_ bond lengths (Ru1-P2 = 2.3717(6) Å; Ru1-P3 = 2.3650(5) Å) than **8**, which are similar to those previously reported for complex **9** (Ru1-P2 = 2.366(1) Å; Ru1-P3 = 2.389(1) Å) and Ru-Zn complex [RuCp(PPh_3_)_2_-*µ*-dmoPTA-1*κP*-2*κ*^2^*N,N*′-ZnCl_2_](CF_3_SO_3_) (Ru1-P2 = 2.377(1) Å; Ru1-P3 = 2.388(1) Å) [24, 27]. The Ru1-S1 bond length in **8** is 2.2764(9) Å, in agreement with analogous Ru(II)-*κS*-DMSO complexes such as [RuCp(DMSO-*κS*)(PPh_3_)(PTA)](CF_3_SO_3_) (2.2616(8) Å) [41].

The palladium atom in **11** is surrounded by the two CH_3_N_dmoPTA_ atoms and two chlorides in a distorted square planar geometry (N1-Pd1-N2 = 88.12(7)^º^; Cl1-Pd1-Cl2 = 85.25(2)^º^; N1-Pd1-Cl1 = 93.68(5)^º^; N2-Pd1-Cl2 = 92.96(5)^º^). The Pd-Cl and Pd-N bond lengths (Pd1-Cl1 = 2.2876(6) Å; Pd1-Cl2 = 2.3059(6) Å; Pd1-N1 = 2.1092(18) Å; Pd1-N2 = 2.1058(19) Å) are close to the typical bond distances in the structure of similar complexes such as *cis*-[PdCl_2_(NH_3_)_2_] (Pd1-Cl1 = 2.26(2) Å; Pd1-Cl2 = 2.29(2) Å; Pd1-N1 = 1.99(4) Å; Pd1-N2 = 2.13(4) Å) [42] and *trans*-[PdCl_2_(NH_3_)_2_] (Pd1-Cl1 = 2.277(2) Å; Pd1-Cl2 = 2.294(2) Å; Pd1-N1 = 2.115(9) Å; Pd1-N2 = 2.097(9) Å) [43]. Finally, the crystal packing of both complexes does not show significant interactions such as *π*-*π* stacking or hydrogen bonding.

### 3.2. Biological Evaluation

#### 3.2.1. Stability Studies

The stability of RuCp complexes in DMSO and in DMSO/H_2_O mixtures has been studied quite extensively in recent years. In general, this family of complexes is reasonably stable in the abovementioned solvent mixtures, and in most cases, decomposition occurs at longer times in comparison with the antiproliferative assays. Nevertheless, typical decomposition pathways usually involve the release of ligands Cl^−^ or PPh_3_, which are substituted by solvent molecules (H_2_O or DMSO) [24, 26, 28].

Complex **8** is soluble in DMSO-d_6_ and DMSO-d_6_/D_2_O (1 : 1) and stable in both media at room temperature and at 37°C as no spectral changes were observed during 48 h (Figures S13–S16 in S.I.). Also, complex **11** is very stable in DMSO-d_6_ at room temperature and 37°C showing two sharp signals relative to the coordinated PPh_3_ groups (39.93 ppm, *d*) and the {dmoPTA-PdCl_2_} unit (−12.07 ppm, *t*) (Figures S17-S18) during 48 h. Nevertheless, a small set of signals corresponding to complex **8** becomes visible after 4 h (<7%), remaining constant up to 48 h. Therefore, only a marginal fraction of **11** undergoes solvolysis in DMSO. In DMSO-d_6_/D_2_O (1 : 1), the ^31^P{^1^H} NMR spectrum of this complex displays broad bands centred at +38.86 ppm assigned to the coordinated PPh_3_ ligands, and different signals, some of them broad, for the {dmoPTA-PdCl_2_} moiety (Figures S19 and S20). These sets of signals may only be explained by the presence of isomers of **11** in dissolution that might be the product of a rapid process involving dissociation and formation of the Pd–N bonds, as observed for previously reported similar complexes [26].

#### 3.2.2. Cytotoxicity Studies

**Scheme 2:**
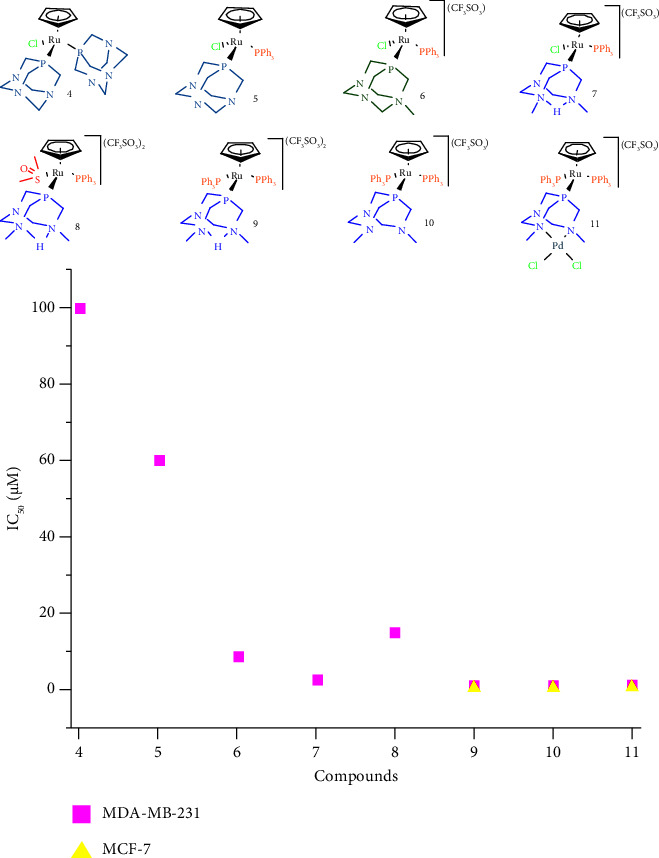
Representation of antiproliferative activity tendency of compounds **4**–**11**.

The cytotoxicity of free-PTA ligands (**1**–**3**) and ruthenium complexes (**4**–**11**) was evaluated using the colorimetric MTT assay against human breast cancer cells MDA-MB-231 and, for the most active compounds, also the MCF-7 cancer cells (Table 1). These cells were selected considering their differences in terms of aggressiveness: the MDA-MB-231 cell line is highly aggressive, invasive, and hormone-independent (estrogen insensitive), while the MCF-7 cell line is noninvasive and hormone-dependent (estrogen sensitive) [46]. The cells were treated with concentrations of compounds in the range of 0.1–100 mM. None of the ligands **1**–**3** were found to be cytotoxic against the evaluated cancer cells (Table 1). In marked contrast, all ruthenium complexes, but **4**, display a notably much better activity (lower IC_50_ value) than cisplatin which was used as the positive control. The results obtained suggest a general trend that was seen for other related ruthenium-cyclopentadienyl compounds: cationic complexes are more cytotoxic than neutral ones (compounds **4-5** vs. **6–10**) [47, 48]. In addition, and as previously observed, a PPh_3_ ligand in the complex structure, but particularly in combination with methylated-PTA, improves cytotoxicity (Scheme 2) [23, 27, 49].

Moderate cytotoxicity is observed for complex **5**, whose structural difference with respect to **4** (noncytotoxic) is the presence of a PPh_3_ ligand instead of one of the two PTA ligands. A 7-fold increase in cytotoxicity with respect to complex **5** is shown, when the PTA ligand is replaced by an mPTA ligand, as in cationic complex **6**. A 4-fold increase in antiproliferative activity can be observed with respect to complex **6**, when HdmoPTA is replaced by an mPTA ligand in the structure of the complex as it happens in **7**. Observing the structural changes in which the antiproliferative activity has increased, complexes **9** and **10** were the most active. Their structures present a combination of PPh_3_ and dimethylated PTA, in place of Cl^−^ and PTA. However, a marked decrease in antiproliferative activity is observed when there is DMSO in place of Cl^−^, as in complex **8**, although the antiproliferative activity is still significant. Nevertheless, and interestingly, the coordination of one {PdCl_2_} moiety to the CH_3_N_dmoPTA_ nitrogen atoms, which gives rise to Ru-Pd heterometallic complex **11**, provides an activity quite similar to those of **9** and **10** [24, 27].

Complexes **9–11**, with the best antiproliferative activity in hormone-independent MDA-MB-231 cells, were further evaluated against the MCF-7 cells, showing even better cytotoxicity activity against this hormone-dependent cancer cell line: IC_50_ values were ∼2-fold lower and very markedly surpassed cisplatin by more than one order of magnitude. In addition, complexes **9**–**11** were tested against normal human dermal fibroblasts (HDF) to evaluate the selectivity for cancer cells. Compared to fibroblasts, complexes **9**, **10**, and **11** are 1.5-fold and ∼2-3-fold less cytotoxic than for MDA-MB-231 cells and MCF-7 cells, respectively. These results are important although not clinically relevant. While further studies are needed, the overall results show that complexes **9–11** seem prospective candidates as metallodrugs for breast cancer showing remarkable activity (in the submicromolar range) against the aggressive, highly metastatic, and cisplatin-resistant MDA-MB-231 line, a model of triple-negative breast cancer, as well as for hormone-dependent MCF-7 cancer cells, largely surpassing the activity of cisplatin by ∼50 fold.

#### 3.2.3. Cell Death Measurement Using Flow Cytometry: The Annexin V/PI Assay

**Figure 3:**
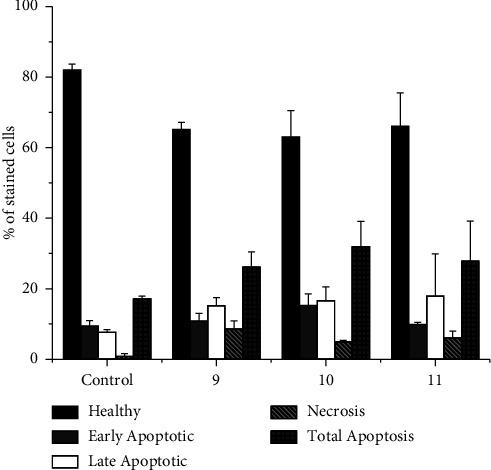
Ruthenium-based compounds potentiate apoptotic cell death in the MDA-MB-231 breast cancer cell line. Apoptotic cell death was analysed by the annexin V fluorescein isothiocyanate (AV-FITC) and propidium iodide (PI) assay in MDA-MB-231 cells, after incubation with IC_50_ concentrations for 24 h graphical representation of the annexin V/PI dot plots of flow cytometry data analysed using Flowing software for control and compounds **9**–**11**.

To determine the cell death mechanism caused by **9**–**11**, the annexin V/propidium iodide (AV/PI) cytometry-based assay was carried out in the MDA-MB-231 cells after 24 h incubation with complexes at their IC_50_ concentrations. Results showed that all compounds mostly induce apoptosis with less than 10% necrosis and that the cells appear at late apoptosis (Figure 3). Induced cell death by apoptosis, a controlled and programmed mechanism of disposal of damaged cells and their content, is a desirable feature for any drug and reinforces the interest of compounds **9**–**11** as prospective therapeutics.

#### 3.2.4. Complex Cell Uptake and Subcellular Distribution

**Figure 4:**
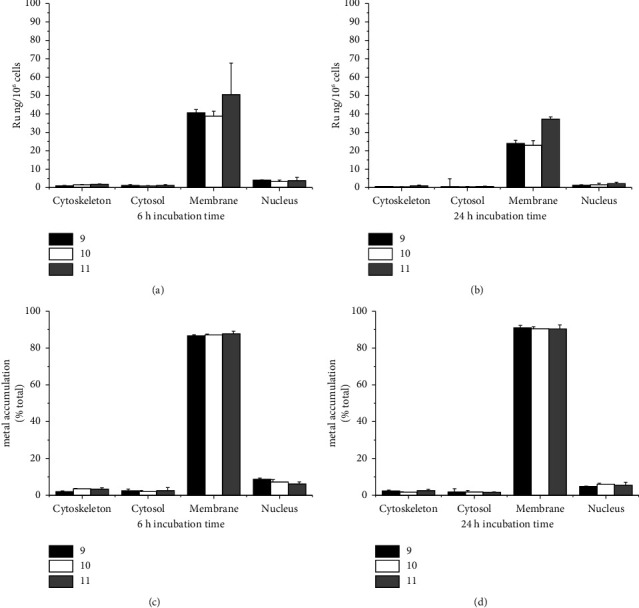
Cellular Ru distribution of **9**–**11** in MDA-MB-23 cells at two different time points: the amount of Ru determined by ICP-MS for each subcellular fraction (normalized, 10^6^ cells) at 6 h (a) and 24 h (b); Ru accumulation (relative %) profile at 6 h (c) and 24 h (d).

The intracellular distribution of complexes **9**–**11** in MDA-MB-231 cells was investigated after 6 h and 24 h incubations at their respective IC_50_ values. Cytosol, membranes, nucleus, and cytoskeletal fractions were extracted using a commercial kit, and the ruthenium content in each fraction was quantified by ICP-MS. Results are summarized in Figure 4.

The results clearly show that all three complexes are mainly accumulated at cell membranes (>86% for 6 h incubation, >90% for 24 h incubation, Figure 4), while ca. 10% or less in the nucleus and marginally in cytosol and cytoskeletal. These results agree with previously published related works on ruthenium-cyclopentadienyl-phosphane-based compounds [44, 50]. No relevant differences in the ruthenium uptake between compounds are observed, possibly due to the very high structural resemblance at the Ru(II) coordination sphere. Despite complex **11** being constituted by Ru and Pd, the observed amount of this last metal in the membranes was only residual, and no evidence of its presence in other organelles of cells was found.

#### 3.2.5. Binding Interaction with Human Serum Albumin by Steady-State and Time-Resolved Fluorescence Spectroscopy

A desirable feature, included in the FDA requirements, for any metallodrug aimed at therapeutic application, is its ability to be transported and distributed within the system where required, at the target sites. This aspect was accessed for complexes **9**–**11**: the possibility of their transport in blood was modelled by the interaction with human serum albumin, the most abundant protein in blood plasma and the major transport vehicle for endo/exogenous compounds in the human system.

Fluorescence spectroscopy was used to assess the interaction between compounds and HSA [50, 51]. HSA exhibits intrinsic fluorescence due to the presence of its phenylalanine, tyrosine, and tryptophan (Trp) residues, of which single Trp at position 214 is the dominant fluorophore [51–55]. Trp214 is located in protein subdomain IIA, near Sudlow's drug binding site I, and it is very sensitive to changes in its microenvironment, which can occur following drug binding in its vicinity or due to structural alterations of protein. Thus, it can also probe interactions at drug-binding site II [56–58]. In this work, we used the intrinsic fluorescence of HSA and selectively monitored the fluorescence emission by Trp214 for constant protein concentration, in the presence of increasing concentrations of the complex [38, 50, 55, 59].

**Figure 5:**
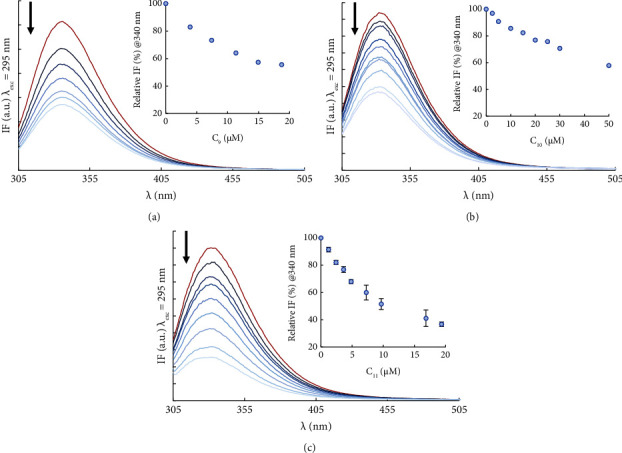
The complexes are effective quenchers of the steady-state fluorescence emission of HSA-Trp214: emission spectra of HSA in the absence (red, top line) and in the presence (blue) of increasing concentrations of **9** (a), **10** (b), and **11** (c) after an incubation of 24 h at 37°C (arrows indicate the change with increasing complex concentration). Insets: the relative fluorescence intensity (%) at *λ*_em_ = 340 nm with increasing complex concentrations, corrected for inner filter effects [40, 60] (conditions: PBS pH 7.4/2% (v/v) DMSO; *C*_HSA_ = 3.7 *µ*M for (a) and 5.0 *µ*M for (b, c); *λ*_exc_ = 295 nm; spectra recorded at 25.0 ± 0.1°C).

*(1) Steady-State and Time-Resolved Fluorescence Emission*. Emission spectra of HSA-Trp214 were acquired in the presence of increasing concentrations of the compounds after an incubation period of 24 h. As can be observed in Figure 5, in the absence of the complex, the maximum emission intensity for Trp214 occurs at 334 nm (red line in Figure 5), in agreement with previous reports [51, 56, 58], and denoting that the indole side chain of Trp214 is not fully exposed to the aqueous solvent, where the maximum emission of Trp would occur at ca. 350 nm [53, 61, 62]. Ru-compounds **9** and **10** do not induce any spectral shift, unlike complex **11**, which causes a small blueshift of the emission by Trp214 (Figure S22). Nonetheless, all complexes caused a marked and concentration-dependent decrease in the Trp214 fluorescence intensity.

The emission intensity of Trp214 after 24 h, at a 20 *µ*M concentration of **9**, **10,** and **11** (corresponding to a 1 : 4 HSA:compound ratio), reached approximately 60%, 75%, and 40%, respectively, of the value measured in the absence of compound (Figure 5, inset). This clear quenching of Trp214 fluorescence indicates that there is a strong interaction between the protein and the three complexes.

The increase in absorbance for the longer wavelengths in the absorption spectra for concentrations higher than 10 *µ*M of **11** is consistent with the formation of an aggregate (Figure S21) [62], which only occurs in the presence of HSA. Thus, high concentrations of **11** may induce HSA aggregation to some extent, which would also explain the previously noted small blueshift of the emission spectra induced by this complex (Figure S22). The global environment of the Trp214 residue would become less polar in the aggregates as it is even more protected from the aqueous environment, leading to the blueshift in emission. In addition, the formation of protein aggregates would also lead to increased light scattering, which is evidenced by the rise in the tail of the absorption spectra of the sample (i.e., an increase in absorbance in a spectral region where none of the species present absorbs light). Nevertheless, it is important to point out that this phenomenon should be of little relevance to the biological effect of the complex because the plasma concentration of HSA is much higher than that used in this study and because the IC_50_ values of **11** are clearly below 10 *µ*M.

**Figure 6:**
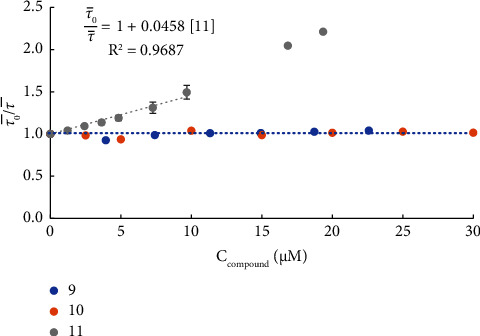
Compound **11**, but not **9** or **10**, affects the amplitude-weighted mean fluorescence lifetime of HSA-Trp214 for a Stern–Volmer plot built from the fluorescence lifetimes of HSA Trp214 in the presence of increasing concentrations of each compound: ratio of the amplitude-weighted mean fluorescence lifetime (see equation (2) in 2.4.6 Interaction with Human Serum Albumin) in the absence ($\bar{\tau }$_0_) and presence ($\bar{\tau }$) of the complex (conditions: PBS pH 7.42% (v/v) DMSO; *C*_HSA_ = 3.7 *µ*M for **9** and 5.0 *µ*M for **10** and **11** *µ*M; *λ*_em_ = 350 nm; the samples were incubated for 24 h at 37°C; the fluorescence intensity decays were recorded at 25.0 ± 0.1°C).

To differentiate multiple binding modes near Trp214 and uncover the quenching mechanism underlying the interaction between HSA and studied complexes, time-resolved fluorescence measurements were carried out. The single-decay analysis of the time-resolved fluorescence of Trp214 measured at different concentrations for each compound revealed a typical triple exponential behaviour [51, 52], e.g., for HSA-Trp214 (Figure S23). For the protein in the absence of the compound, the amplitudes were *α*_1_ = 0.34 ± 0.01, *α*_2_ = 0.41 ± 0.01, and *α*_3_ = 0.25 ± 0.01; the lifetime components were *τ*_1_ = (1.03 ± 0.13) ns, *τ*_2_ = (3.95 ± 0.13) ns, and *τ*_3_ = (7.31 ± 0.01) ns; and the amplitude-weighted mean fluorescence lifetime, $\bar{\tau }$, was (3.80 ± 0.15) ns, all in agreement with previous reports [51, 52]. With the calculated amplitude-weighted mean fluorescence lifetime values, it is possible to represent a fluorescence lifetime Stern–Volmer plot (Figure 6).

Figure 6 shows that the presence of **9** and **10** does not affect the mean fluorescence lifetime of HSA-Trp214 regardless of the concentration used. This fact indicates that these complexes quench the steady-state fluorescence of HSA without affecting its fluorescence lifetime. This result points to the formation of a nonfluorescent ground state complex through the binding of **9** and **10** to the protein near the Trp214 residue.

In contrast, the amplitude-weighted mean fluorescence lifetime of Trp214 decreased in the presence of **11**, which suggests that dynamic quenching might be occurring. Typical Stern–Volmer constants are in the order of 10 mM–100 mM for dynamic quenching resulting from random collisions between the compound and the indole sidechain of Trp214; however, for the concentration range here used, there could be no appreciable collisional quenching. Therefore, the observed behaviour should mean that fluorescence lifetime is due to a stronger and more specific interaction related to a binding process. As both amplitude-weighted mean fluorescence lifetimes decrease to a larger extent in the steady-state fluorescence intensity, the presence of two independent binding sites should be considered: one of them in closer proximity to Trp214 than the other. However, more complex situations cannot be completely ruled out, such as two binding modes in the same binding site or two binding sites, both close to Trp214.

**Figure 7:**
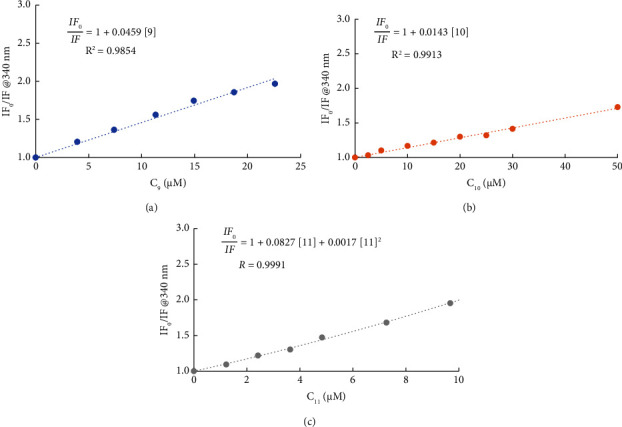
HSA-Trp214 steady-state fluorescence Stern–Volmer plots for quenching by the compounds. Variation in the fluorescence intensity of Trp214 at 340 nm obtained from steady-state measurements (corrected for inner filter and self-absorption effects) in the absence (IF_0_) and presence (IF) of compounds **9** (a), **10** (b), and **11** (c) (conditions: PBS pH 7.4/2% (v/v) DMSO; *C*_HSA_ = 3.7 *µ*M for **9** and 5.0 *µ*M for **10** and **11**, kept constant; *λ*_exc_ = 295 nm, *λ*_em_ = 340 nm; the samples were incubated for 24 h at 37°C; spectra were recorded at 25.0 ± 0.1°C).

*(2) Determination of the Compound-HSA-Binding Constants*. The first approach to estimate the complex-HSA-binding constants based on fluorescence quenching is usually the inspection of the Stern–Volmer plots. No variation in the amplitude-weighted mean fluorescence lifetime was detected for compounds **9** and **10**. However, this was not the case for the steady-state fluorescence intensity-based Stern–Volmer plots which are shown in Figure 7.

Considering the absence of the effect on the fluorescence lifetimes for **9** and **10**, the slope of the Stern–Volmer plots in Figures 7(a) and 7(b) can be safely interpreted as a static quenching constant. In turn, in these two specific situations, the static quenching constant for Trp214 by each compound can be taken as the binding constant (*K*_*B*_) for the formation of a ground state 1 : 1 nonfluorescent complex between HSA and the compound (equation (4)), according to the equilibrium:

(3) $$\begin{array}{c} {\left\{HSA\right\}}_{\left(aq\right)}+{\left\{compound\right\}}_{\left(aq\right)}⇄{\left\{HSA–compound\right\}}_{\left(aq\right)}\text{,} \end{array}$$

(4) $$\begin{array}{c} \frac{{IF}_{0}}{IF}=1+K_{B}C_{compound,} \end{array}$$

where IF_0_ and IF are, respectively, the fluorescence intensity of Trp214 of HSA in the absence and presence of the compound. The determined binding constant for **9** of (4.59 ± 0.21) × 10^4^·M^−1^ and of (1.43 ± 0.05) × 10^4^·M^−1^ for **10** correspond to log *K*_*B*_ values of (4.66 ± 0.04) for **9** and (4.16 ± 0.03) for **10**, respectively.

From a linear fit to the amplitude-weighted mean fluorescence lifetime of the Stern–Volmer plot for HSA-Trp214 quenching by **11** (Figure 6), it is possible to estimate conditional binding constant *K*′_*B*_ of (4.58 ± 0.33) × 10^4^·M^−1^. The two quenching processes, affecting the steady-state fluorescence intensity alone or together with the fluorescence lifetime, strongly suggest two binding modes as previously noted. Therefore, the following equation was fitted to the variation of the steady-state fluorescence intensity of Trp214 with an increasing concentration of **11** (Figure 7(c)):

(5) $$\begin{array}{c} \frac{{IF}_{0}}{IF}\approx 1+\left(K_{B}+K_{B}^{\prime }\right)C_{11}+\left(K_{B}K_{B}^{\prime }\right){\left(C_{11}\right)}^{2}\text{,} \end{array}$$

where *K′*_*B*_ was fixed to (4.58 ± 0.33) × 10^4^ M^−1^. A *K*_*B*_ of (3.70 ± 0.33) × 10^4^ M^−1^ was retrieved, corresponding to a log *K*_*B*_ of (4.57 ± 0.07). Moreover, as previously stated, *K′*_*B*_ can be considered an additional binding constant for the equilibrium between **11** and HSA, being log *K′*_*B*_ of (4.66 ± 0.07). Interestingly, both *K*_*B*_ and *K′*_*B*_ are in the same order of magnitude as all other binding constants determined. Note that concentrations of **11** above 10 *µ*M were not considered for the estimation of either *K*_*B*_ or *K′*_*B*_ (Figures 6 and 7(c)), due to the possible aggregation of HSA, which was reported in the previous section.

Table 2 presents the summary of all the binding constants determined for the interaction of HSA with **9**, **10**, and **11**.

The *K*_B_ values obtained for the interaction of HSA with each compound are very similar. Also, the strong interaction of ruthenium [51] and copper complexes [58, 63] with HSA determined in previous works is comparable with the values of binding constants obtained herein for all three compounds. Interestingly, heterodimetallic complex **11** is the one with the strongest interaction with HSA, due to the two modes of interaction that are relatively efficient. Monometallic parent complexes **9** and finally **10** show a similar interaction with HSA, which is smaller than that observed for **11**. Log *K*_*B*_ obtained for complexes **9**–**11** are of the same order of magnitude as the one found for the Ru(III) complex KP1019, known to be transported in blood by serum albumin and to bind the protein reversibly [57]. Our results thus suggest that all three compounds, **9**, **10** and **11**, can be efficiently transported by albumin in blood plasma.

## 4. Conclusions

Complex [RuCp(DMSO-*κS*)(HdmoPTA)(PPh_3_)](CF_3_SO_3_)_2_ (**8**) and heterodimetallic [RuCp(PPh_3_)_2_(dmoPTA-1*κP*-2*κ*^2^*N,N*′-PdCl_2_)](CF_3_SO_3_) (**11**) were characterized by elemental analysis, IR, and multinuclear NMR, and their crystal structures were determined by single-crystal X-ray diffraction. Also, the stability of these complexes in DMSO and DMSO/D_2_O was performed, resulting in **8** formed in low proportion (<7%) when **11** is dissolved in pure DMSO.

Not only the cytotoxic activities of all the complexes (**4**–**11**) but also those of ligands PTA (**1**), mPTA (**2**), and dmPTA (**3**) were evaluated against the MDA-MB-231 and MCF-7 breast cancer cells using the MTT assay. The most active complexes against both breast cancer cells were **9**, **10**, and **11**, showing IC_50_ values in the micromolar range, suggesting that the highest cytotoxicity is achieved when two PPh_3_ ligands and one dmoPTA ligand were combined around the metal in the {CpRu} moiety. These complexes were also tested against normal human dermal fibroblasts (HDFs) to evaluate the selectivity for cancer cells, being 1.5-fold and ∼2-3-fold less cytotoxic than for MDA-MB-231 cells and MCF-7 cells, respectively. Studies by flow cytometry showed that these three complexes induce mostly apoptosis in combination with a small percentage of necrosis (<10%) on MDA-MB-231 cells, which is very positive for their possible use as a drug. Cell distribution studies of the three complexes showed that Ru is mostly retained in the cell membrane (ca. 90%) and a reduced quantity in the cell nucleus (<10%) of MDA-MB-231 cells. It is important to stress that Pd metal was not found in a significant quantity in cells when **11** was evaluated. Experiments to determine the binding interaction with human serum albumin were carried out, concluding that the presence or absence of an H-bridge between CH_3_N_dmoPTA_ determines the interaction mechanism with the carrier blood protein. Also, an interesting conclusion is that Ru-Pd heterobimetallic complex **11** interacts with HSA to a higher extent and by a different mechanism with respect to monometallic complexes. The obtained results show how the combination of ligands around Ru determines the antiproliferative activity of this family of complexes, but the fact that Ru is located mainly in the cell membrane suggests that these ligands should affect how the cell membrane works, being the factor that induces their cytotoxic activity. The fact that Pd is not located in the cell also suggests that complex **11** decomposes, releasing the Pd atom before interacting with the cells, but the fact that this complex interacts with HSA by a different mechanism than **9** and **10** also suggests that this extra metal can determine how the complex arrives to the cell, being this also an important role related to the antiproliferative activity. Additional experiments are in progress to determine these suspicions. Overall, complexes **9** and **10** emerge from this work as interesting and highly promising to pursue to further studies as prospective ruthenium metallodrugs. 

## Figures and Tables

**Table 1:** Cytotoxicity studies.

Compounds						MDA-MB-231	MCF-7	HDF
Cisplatin [44, 45]	—	—		—	—	>100	38 ± 1.4	∼100

**1**	PTA				0	>100	—	—
**2**	mPTA				+1	>100	—	—
**3**	dmPTA				+2	>100	—	—

Structure [Ru(Cp)*L*^1^*L*^2^*L*^3^]	*L* ^1^		*L* ^2^	*L* ^3^	Charge			

**4**	Cl^−^		PTA	PTA	0	>100	—	—
**5**	Cl^−^		PPh_3_	PTA	0	60 ± 19	—	—
**6**	Cl^−^		PPh_3_	mPTA	+1	8.3 ± 1.4	—	—
7	Cl^−^		PPh_3_	HdmoPTA	+1	2.5 ± 1.1	—	—
**8**	κ*S*-DMSO		PPh_3_	HdmoPTA	+2	15 ± 2.2	—	—
**9**	PPh_3_		PPh_3_	HdmoPTA	+2	0.86 ± 0.13	0.48 ± 0.07	1.23 ± 0.32
**10**	PPh_3_		PPh_3_	dmoPTA	+1	0.84 ± 0.09	0.40 ± 0.04	1.47 ± 0.40
**11**	PPh_3_		PPh_3_	dmoPTA-PdCl_2_	+1	1.1 ± 0.2	0.73 ± 0.11	1.59 ± 0.45

The IC_50_ values (*µ*M) found after 24 h incubation of compounds **1**–**11** with MDA-MB-231 and of compounds **9**, **10**, and **11** with MCF-7 breast cancer cells and normal human fibroblasts (HDF). Values are the average of at least three experiments (±SD).

**Table 2:** Parameters determined for the binding of **9**, **10**, and **11** with HSA.

Compound	Type of quenching	Equation used	*K*_*B*_ × 10^4^ (M^−1^)	*K′*_*B*_ × 10^4^ (M^−1^)	Log *K*_*B*_	Log *K′*_*B*_
**9**	Static	3	4.59 ± 0.21	—	4.66 ± 0.04	—
**10**	Static	3	1.43 ± 0.05	—	4.16 ± 0.03	—
**11**	“Dynamic” and static	4	3.70 ± 0.33	4.58 ± 0.33	4.57 ± 0.07	4.66 ± 0.07

Conditions: PBS pH 7.4/2% (v/v) DMSO; *C*_HSA_ = 3.7 *µ*M for **9** and 5.0 *µ*M for **10** and **11** *µ*M, kept constant; the samples were incubated for 24 h at 37°C.

## Data Availability

Crystallographic data for the structures reported in this manuscript have been deposited with the Cambridge Crystallographic Data Centre under the CCDC codes 2263437 and 2261422. These data can be obtained free of charge via http://www.ccdc.cam.ac.uk/data_request/cif, or by emailing data_request@ccdc.cam.ac.uk, or by contacting the Cambridge Crystallographic Data Centre, 12 Union Road, Cambridge CB2 1EZ, UK; fax: +44 1223 336033.

## Supplementary Materials

Supplementary Materials Supplementary Materials contains experimental procedures, NMR data, X-ray crystallographic data for **8** and **11**, and antiproliferative and biological studies: stability tests in DMSO-d_6_ and DMSO-d_6_/D_2_O under air atmosphere; cell lines and culture conditions; compound cytotoxicity evaluated using the MTT assay; cell death measurement using flow cytometry: the annexin V/PI assay; complex uptake and distribution by ICP-MS; binding interaction with human serum albumin by steady-state and time-resolved fluorescence emission. Also, separate check cif files (PDF) for crystal structures of **8** and **11** were provided.
