# Supplementary material for: Evaluation of the Antiproliferative Properties of CpRu Complexes Containing N-Methylated Triazaphosphaadamantane Derivatives
Source: Bioinorg Chem Appl. 2023 Sep 28;2023:6669394. doi: 10.1155/2023/6669394 (PMC10555500; doi:10.1155/2023/6669394)
Supplement: Supplementary Materials — contains experimental procedures, NMR data, X-ray crystallographic data for 8 and 11, and antiproliferative and biological studies: stability tests in DMSO-d6 and DMSO-d6/D2O under air atmosphere; cell lines and culture conditions; compound cytotoxicity evaluated using the MTT assay; cell death measurement using flow cytometry: the annexin V/PI assay; complex uptake and distribution by ICP-MS; binding interaction with human serum albumin by steady-state and time-resolved fluorescence emission. Also, separate check cif files (PDF) for crystal structures of 8 and 11 were provided. [file 6669394.f1.zip › 2261422_checkcif.pdf]

No syntax errors found.
Please wait while processing ....

CIF dictionary
Interpreting this report

Datablock: mo_aa444_0ma

Bond precision:	C-C = 0.0037 A		Wavelength=0.71073
Cell:	a=12.8359(7)	b=14.4650(8)	c=19.5733(12)
	alpha=92.140(2)	beta=104.143(2)	gamma=111.894(1)
Temperature :	100 K		

	Calculated	Reported
Volume	3236.4(3)	3236.4(3)
Space group	P -1	P -1
Hall group	-P 1	-P 1
Moiety formula	C48 H51 Cl2 N3 P3 Pd Ru, C F3 O3 S, 4(C H Cl3)	C48 H51 Cl2 N3 P3 Pd Ru, C F3 O3 S, 4(C H Cl3)
Sum formula	C53 H55 Cl14 F3 N3 O3 P3 Pd Ru S	C53 H55 Cl14 F3 N3 O3 P3 Pd Ru S
Mr	1667.77	1667.74
Dx,g cm-3	1.711	1.711
Z	2	2
Mu (mm-1)	1.249	1.249
F000	1668.0	1668.0
F000'	1668.98	
h,k,lmax	18,20,27	18,20,27
Nref	18943	18933
Tmin,Tmax	0.861,0.883	0.656,0.747
Tmin'	0.687	

Correction method= # Reported T Limits: Tmin=0.656 Tmax=0.747 AbsCorr = MULTI-SCAN

Data completeness= 0.999 Theta(max)= 30.034

R(reflections)= 0.0360(17788) wR2(reflections)= 0.0761(18933)

S = 1.145 Npar= 921

The following ALERTS were generated. Each ALERT has the format
test-name_ALERT_alert-type_alert-level.
Click on the hyperlinks for more details of the test.

Alert level C

```
PLAT220_ALERT_2_C NonSolvent   Resd 1   C   Ueq(max)/Ueq(min) Range        3.2 Ratio
PLAT244_ALERT_4_C Low     'Solvent' Ueq as Compared to Neighbors of        S1T Check
PLAT244_ALERT_4_C Low     'Solvent' Ueq as Compared to Neighbors of        C1C Check
PLAT336_ALERT_2_C Long Bond Distance for ..... C2C      -Cl14            1.930 Ang.
PLAT906_ALERT_3_C Large K Value in the Analysis of Variance ......       2.701 Check
PLAT910_ALERT_3_C Missing # of FCF Reflection(s) Below Theta(Min).           6 Note
PLAT911_ALERT_3_C Missing FCF Refl Between Thmin & STh/L=    0.600           4 Report
PLAT977_ALERT_2_C Check Negative Difference Density on H3CA      .       -0.31 eA-3
```

Alert level G

```
PLAT002_ALERT_2_G Number of Distance or Angle Restraints on AtSite           4 Note
PLAT012_ALERT_1_G N.O.K.   _shelx_res_checksum Found in CIF ......     Please Check
PLAT083_ALERT_2_G SHELXL Second Parameter in WGHT  Unusually Large        7.44 Why ?
PLAT171_ALERT_4_G The CIF-Embedded .res File Contains EADP Records           2 Report
PLAT172_ALERT_4_G The CIF-Embedded .res File Contains DFIX Records           6 Report
PLAT231_ALERT_4_G Hirshfeld Test (Solvent)   Cl11     --C2C      .        17.5 s.u.
```

And 2 other PLAT231 Alerts

```
PLAT231_ALERT_4_G Hirshfeld Test (Solvent)   Cl12     --C2C      .        14.5 s.u.
PLAT231_ALERT_4_G Hirshfeld Test (Solvent)   Cl14     --C2C      .         9.0 s.u.
```

```
PLAT232_ALERT_2_G Hirshfeld Test Diff (M-X)  Ru1      --P2       .         5.5 s.u.
PLAT302_ALERT_4_G Anion/Solvent/Minor-Residue Disorder (Resd  2  )         88% Note
```

And 6 other PLAT302 Alerts

```
PLAT302_ALERT_4_G Anion/Solvent/Minor-Residue Disorder (Resd  3  )        75% Note
PLAT302_ALERT_4_G Anion/Solvent/Minor-Residue Disorder (Resd  4  )       100% Note
PLAT302_ALERT_4_G Anion/Solvent/Minor-Residue Disorder (Resd  5  )       100% Note
PLAT302_ALERT_4_G Anion/Solvent/Minor-Residue Disorder (Resd  7  )       100% Note
PLAT302_ALERT_4_G Anion/Solvent/Minor-Residue Disorder (Resd  8  )       100% Note
PLAT302_ALERT_4_G Anion/Solvent/Minor-Residue Disorder (Resd  9  )       100% Note


PLAT304_ALERT_4_G Non-Integer Number of Atoms in ..... (Resd  4  )       2.61 Check
And 4 other PLAT304 Alerts

PLAT304_ALERT_4_G Non-Integer Number of Atoms in ..... (Resd  5  )       3.87 Check
PLAT304_ALERT_4_G Non-Integer Number of Atoms in ..... (Resd  7  )       1.64 Check
PLAT304_ALERT_4_G Non-Integer Number of Atoms in ..... (Resd  8  )       0.76 Check
PLAT304_ALERT_4_G Non-Integer Number of Atoms in ..... (Resd  9  )       1.13 Check


PLAT432_ALERT_2_G Short Inter X...Y Contact  Cl16     ..C12       .       3.23 Ang.
                                              1-x,2-y,1-z  =      2_676 Check
And 2 other PLAT432 Alerts

PLAT432_ALERT_2_G Short Inter X...Y Contact  Cl17     ..C30       .       3.08 Ang.
                                                  x,1+y,z  =      1_565 Check
PLAT432_ALERT_2_G Short Inter X...Y Contact  Cl5C     ..C24       .       3.19 Ang.
                                              1-x,1-y,1-z  =      2_666 Check
And 2 other PLAT432 Alerts

PLAT432_ALERT_2_G Short Inter X...Y Contact  Cl19     ..C5        .       3.18 Ang.
                                                    x,y,z  =      1_555 Check
PLAT432_ALERT_2_G Short Inter X...Y Contact  Cl20     ..C24       .       3.15 Ang.
                                              1-x,2-y,1-z  =      2_676 Check
PLAT720_ALERT_4_G Number of Unusual/Non-Standard Labels ..........         25 Note
PLAT790_ALERT_4_G Centre of Gravity not Within Unit Cell: Resd.  #          5 Note
              C H Cl3
PLAT790_ALERT_4_G Centre of Gravity not Within Unit Cell: Resd.  #          9 Note
              C H Cl3
PLAT794_ALERT_5_G Tentative Bond Valency for Pd1       (II)       .       1.91 Info
PLAT860_ALERT_3_G Number of Least-Squares Restraints .............          7 Note
PLAT912_ALERT_4_G Missing # of FCF Reflections Above STh/L=  0.600          1 Note
PLAT913_ALERT_3_G Missing # of Very Strong Reflections in FCF ....          1 Note
PLAT933_ALERT_2_G Number of HKL-OMIT Records in Embedded .res File          1 Note
PLAT978_ALERT_2_G Number C-C Bonds with Positive Residual Density.          2 Info
```

```
   0 ALERT level A = Most likely a serious problem - resolve or explain
   0 ALERT level B = A potentially serious problem, consider carefully
   8 ALERT level C = Check. Ensure it is not caused by an omission or oversight
  35 ALERT level G = General information/check it is not something unexpected

   1 ALERT type 1 CIF construction/syntax error, inconsistent or missing data
  13 ALERT type 2 Indicator that the structure model may be wrong or deficient
   5 ALERT type 3 Indicator that the structure quality may be low
  23 ALERT type 4 Improvement, methodology, query or suggestion
   1 ALERT type 5 Informative message, check
```

It is advisable to attempt to resolve as many as possible of the alerts in all categories. Often the minor alerts point to easily fixed oversights, errors and omissions in your CIF or refinement strategy, so attention to these fine details can be worthwhile. In order to resolve some of the more serious problems it may be necessary to carry out additional measurements or structure refinements. However, the purpose of your study may justify the reported deviations and the more serious of these should normally be commented upon in the discussion or experimental section of a paper or in the "special_details" fields of the CIF. checkCIF was carefully designed to identify outliers and unusual parameters, but every test has its limitations and alerts that are not important in a particular case may appear. Conversely, the absence of alerts does not guarantee there are no aspects of the results needing attention. It is up to the individual to critically assess their own results and, if necessary, seek expert

advice.

Publication of your CIF in IUCr journals

A basic structural check has been run on your CIF. These basic checks will be run on all CIFs submitted for publication in IUCr journals (*Acta Crystallographica*, *Journal of Applied Crystallography*, *Journal of Synchrotron Radiation*); however, if you intend to submit to *Acta Crystallographica Section C* or *E* or *IUCrData*, you should make sure that full publication checks are run on the final version of your CIF prior to submission.

Publication of your CIF in other journals

Please refer to the *Notes for Authors* of the relevant journal for any special instructions relating to CIF submission.

PLATON version of 10/05/2023; check.def file version of 10/05/2023

Datablock mo_aa444_0ma - ellipsoid plot

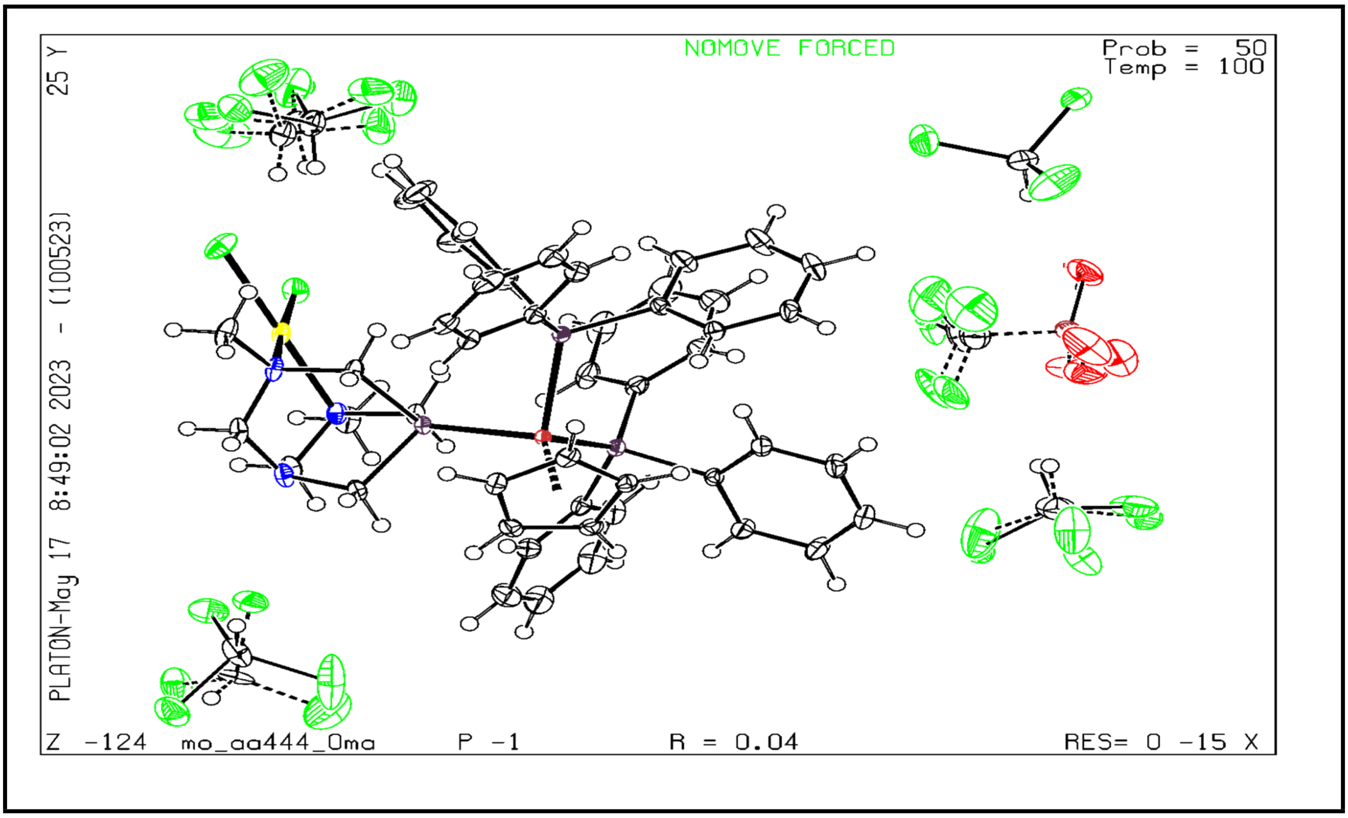

Download CIF editor (publCIF) from the IUCr
Download CIF editor (enCIFer) from the CCDC
Test a new CIF entry
