# Supplementary material for: Evaluation of the Antiproliferative Properties of CpRu Complexes Containing N-Methylated Triazaphosphaadamantane Derivatives
Source: Bioinorg Chem Appl. 2023 Sep 28;2023:6669394. doi: 10.1155/2023/6669394 (PMC10555500; doi:10.1155/2023/6669394)
Supplement: Supplementary Materials — contains experimental procedures, NMR data, X-ray crystallographic data for 8 and 11, and antiproliferative and biological studies: stability tests in DMSO-d6 and DMSO-d6/D2O under air atmosphere; cell lines and culture conditions; compound cytotoxicity evaluated using the MTT assay; cell death measurement using flow cytometry: the annexin V/PI assay; complex uptake and distribution by ICP-MS; binding interaction with human serum albumin by steady-state and time-resolved fluorescence emission. Also, separate check cif files (PDF) for crystal structures of 8 and 11 were provided. [file 6669394.f1.zip › 2263437_checkcif.pdf]

No syntax errors found.
Please wait while processing ....

CIF dictionary
Interpreting this report

Datablock: mo_aaa_fr_0m

Bond precision:	C-C = 0.0066 A		Wavelength=0.71073
Cell:	a=9.5483(2)	b=11.6728(2)	c=40.0824(8)
	alpha=90	beta=95.371(1)	gamma=90
Temperature :	302 K		

	Calculated	Reported
Volume	4447.79(15)	4447.79(15)
Space group	P 21/c	P 1 21/c 1
Hall group	-P 2ybc	-P 2ybc
Moiety formula	C32 H43 N3 O P2 Ru S, 2(C F3 O3 S), C3 H6 O	C32 H43 N3 O P2 Ru S, 2(C F3 O3 S), C3 H6 O
Sum formula	C37 H49 F6 N3 O8 P2 Ru S3	C37 H49 F6 N3 O8 P2 Ru S3
Mr	1036.98	1036.98
Dx,g cm-3	1.549	1.549
Z	4	4
Mu (mm-1)	0.642	0.642
F000	2128.0	2128.0
F000'	2126.34	
h,k,lmax	14,17,58	14,17,58
Nref	14789	14781
Tmin,Tmax	0.781,0.825	0.066,0.102
Tmin'	0.766	

Correction method= # Reported T Limits: Tmin=0.066 Tmax=0.102 AbsCorr = MULTI-SCAN
Data completeness= 0.999 Theta(max)= 31.506
R(reflections)= 0.0622(11828) wR2(reflections)= 0.1620(14781)
S = 1.061 Npar= 551

The following ALERTS were generated. Each ALERT has the format
test-name_ALERT_alert-type_alert-level.
Click on the hyperlinks for more details of the test.

Alert level C

```
PLAT241_ALERT_2_C High    'MainMol' Ueq as Compared to Neighbors of        C16 Check
PLAT242_ALERT_2_C Low     'MainMol' Ueq as Compared to Neighbors of        Ru1 Check
PLAT244_ALERT_4_C Low     'Solvent' Ueq as Compared to Neighbors of        S1T Check
PLAT244_ALERT_4_C Low     'Solvent' Ueq as Compared to Neighbors of        C2A Check
PLAT260_ALERT_2_C Large Average Ueq of Residue Including       S1T      0.113 Check
PLAT906_ALERT_3_C Large K Value in the Analysis of Variance ......      6.427 Check
PLAT911_ALERT_3_C Missing FCF Refl Between Thmin & STh/L=     0.600         6 Report
PLAT913_ALERT_3_C Missing # of Very Strong Reflections in FCF ....          4 Note
PLAT976_ALERT_2_C Check Calcd Resid. Dens.  0.47Ang From O5TA     .     -0.54 eA-3
```

Alert level G

```
PLAT007_ALERT_5_G Number of Unrefined Donor-H Atoms ..............          1 Report
PLAT083_ALERT_2_G SHELXL Second Parameter in WGHT  Unusually Large       8.15 Why ?
PLAT171_ALERT_4_G The CIF-Embedded .res File Contains EADP Records          5 Report
PLAT187_ALERT_4_G The CIF-Embedded .res File Contains RIGU Records          1 Report
PLAT231_ALERT_4_G Hirshfeld Test (Solvent)    S2TA     --O4TA      .     11.3 s.u.
```

And 10 other PLAT231 Alerts

```
PLAT231_ALERT_4_G Hirshfeld Test (Solvent)    S2TA     --O6TA      .     11.0 s.u.
PLAT231_ALERT_4_G Hirshfeld Test (Solvent)    S2TA     --O5TA      .     15.0 s.u.
PLAT231_ALERT_4_G Hirshfeld Test (Solvent)    S2TB     --C2T       .      6.4 s.u.
PLAT231_ALERT_4_G Hirshfeld Test (Solvent)    F4TA     --C2T       .      5.4 s.u.
PLAT231_ALERT_4_G Hirshfeld Test (Solvent)    F6TA     --C2T       .      6.4 s.u.
PLAT231_ALERT_4_G Hirshfeld Test (Solvent)    F5TA     --C2T       .      5.6 s.u.
PLAT231_ALERT_4_G Hirshfeld Test (Solvent)    S1T      --C1T       .      6.2 s.u.
PLAT231_ALERT_4_G Hirshfeld Test (Solvent)    F1T      --C1T       .      7.7 s.u.
```

```
PLAT231_ALERT_4_G Hirshfeld Test (Solvent)    F2T      --C1T      .        9.2 s.u.
PLAT231_ALERT_4_G Hirshfeld Test (Solvent)    F3T      --C1T      .       14.0 s.u.


PLAT244_ALERT_4_G Low    'Solvent' Ueq as Compared to Neighbors of        C1T Check
PLAT302_ALERT_4_G Anion/Solvent/Minor-Residue Disorder (Resd  2  )        88% Note
PLAT720_ALERT_4_G Number of Unusual/Non-Standard Labels ..........         20 Note
PLAT860_ALERT_3_G Number of Least-Squares Restraints .............        511 Note
PLAT910_ALERT_3_G Missing # of FCF Reflection(s) Below Theta(Min).          2 Note
PLAT978_ALERT_2_G Number C-C Bonds with Positive Residual Density.          0 Info
PLAT992_ALERT_5_G Repd & Actual _reflns_number_gt Values Differ by          2 Check
```

```
   0 ALERT level A = Most likely a serious problem - resolve or explain
   0 ALERT level B = A potentially serious problem, consider carefully
   9 ALERT level C = Check. Ensure it is not caused by an omission or oversight
  22 ALERT level G = General information/check it is not something unexpected

   0 ALERT type 1 CIF construction/syntax error, inconsistent or missing data
   6 ALERT type 2 Indicator that the structure model may be wrong or deficient
   5 ALERT type 3 Indicator that the structure quality may be low
  18 ALERT type 4 Improvement, methodology, query or suggestion
   2 ALERT type 5 Informative message, check
```

It is advisable to attempt to resolve as many as possible of the alerts in all categories. Often the minor alerts point to easily fixed oversights, errors and omissions in your CIF or refinement strategy, so attention to these fine details can be worthwhile. In order to resolve some of the more serious problems it may be necessary to carry out additional measurements or structure refinements. However, the purpose of your study may justify the reported deviations and the more serious of these should normally be commented upon in the discussion or experimental section of a paper or in the "special_details" fields of the CIF. checkCIF was carefully designed to identify outliers and unusual parameters, but every test has its limitations and alerts that are not important in a particular case may appear. Conversely, the absence of alerts does not guarantee there are no aspects of the results needing attention. It is up to the individual to critically assess their own results and, if necessary, seek expert advice.

Publication of your CIF in IUCr journals

A basic structural check has been run on your CIF. These basic checks will be run on all CIFs submitted for publication in IUCr journals (*Acta Crystallographica*, *Journal of Applied Crystallography*, *Journal of Synchrotron Radiation*); however, if you intend to submit to *Acta Crystallographica Section C* or *E* or *IUCrData*, you should make sure that full publication checks are run on the final version of your CIF prior to submission.

Publication of your CIF in other journals

Please refer to the *Notes for Authors* of the relevant journal for any special instructions relating to CIF submission.

PLATON version of 10/05/2023; check.def file version of 10/05/2023

Datablock mo_aaa_fr_0m - ellipsoid plot

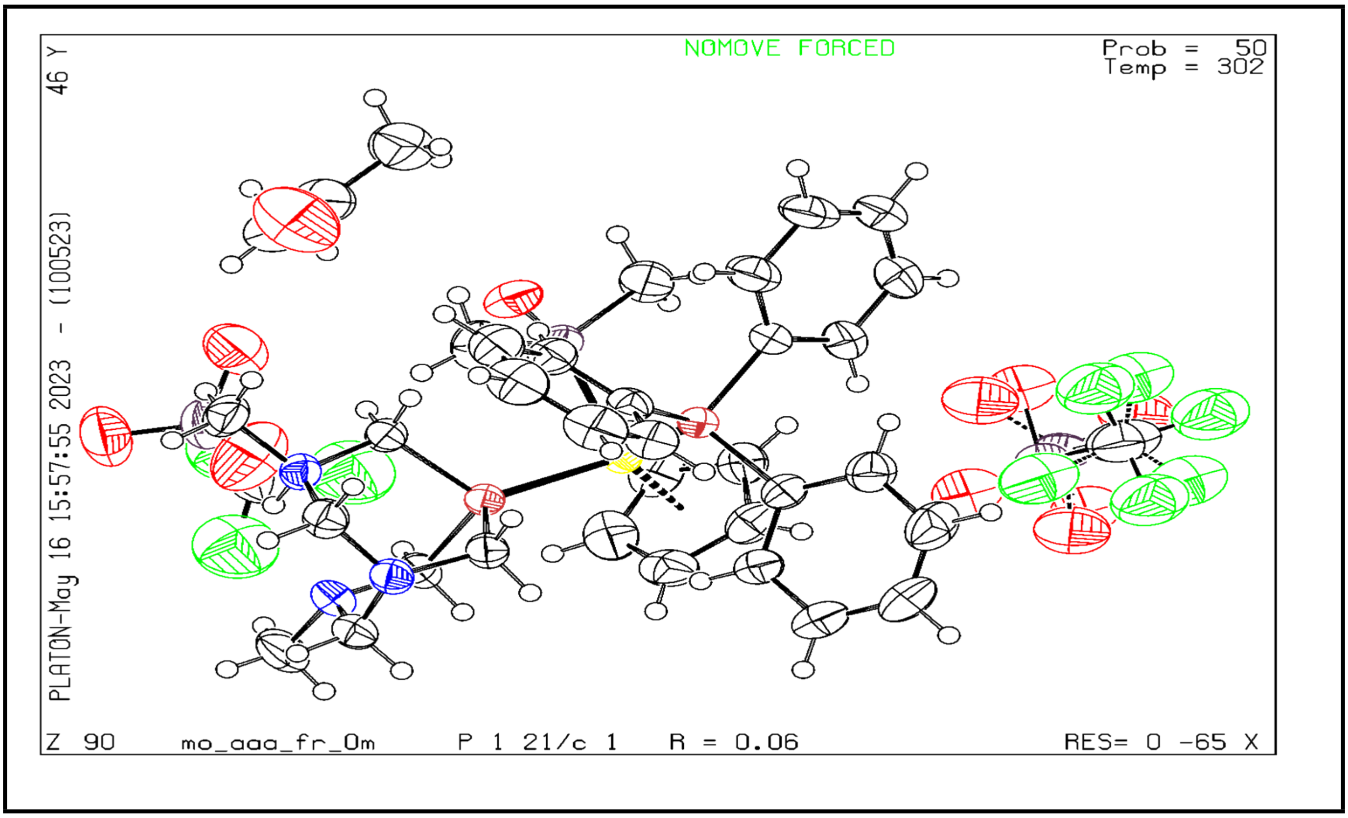

Download CIF editor (publCIF) from the IUCr
Download CIF editor (enCIFer) from the CCDC
Test a new CIF entry
