# Supplementary material for: Evaluation of the Antiproliferative Properties of CpRu Complexes Containing N-Methylated Triazaphosphaadamantane Derivatives
Source: Bioinorg Chem Appl. 2023 Sep 28;2023:6669394. doi: 10.1155/2023/6669394 (PMC10555500; doi:10.1155/2023/6669394)
Supplement: Supplementary Materials — contains experimental procedures, NMR data, X-ray crystallographic data for 8 and 11, and antiproliferative and biological studies: stability tests in DMSO-d6 and DMSO-d6/D2O under air atmosphere; cell lines and culture conditions; compound cytotoxicity evaluated using the MTT assay; cell death measurement using flow cytometry: the annexin V/PI assay; complex uptake and distribution by ICP-MS; binding interaction with human serum albumin by steady-state and time-resolved fluorescence emission. Also, separate check cif files (PDF) for crystal structures of 8 and 11 were provided. [file 6669394.f1.zip › SI_PTA_Ru_Lisbon-BCaA-3.3.docx]

Supplementary Materials

Evaluation of the antiproliferative properties of CpRu complexes containing N-methylated triazaphosphaadamantane derivatives

Andrés Alguacil,^a^ Franco Scalambra,^a^ Antonio Romerosa,^a^ Andreia Bento-Oliveira,^b^ Fernanda Marques,^c^ Inês Maximiano,^b^ Rodrigo F. M. de Almeida,^b^ Ana Isabel Tomaz,^b^ Andreia Valente.^b^

*^a^ Área de Química Inorgánica – CIESOL, Universidad de Almería, 04120 Almería, Spain*

*^b^ Centro de Química Estrutural, Institute of Molecular Sciences, Departamento de Química e Bioquímica, Faculdade de Ciências, Universidade de Lisboa, Campo Grande, 1749-016 Lisboa, Portugal.*

*^c^ Centro de Ciências e Tecnologias Nucleares e Departamento de Engenharia e Ciências Nculeares, Instituto Superior Técnico (C2TN/IST), Universidade de Lisboa, Estrada Nacional 10, 2695-066, Bobadela LRS, Portugal*

Correspondence should be addressed mainly to Antonio Romerosa; [romerosa@ual.es](mailto:romerosa@ual.es) ;

biological procedure issues to Andreia Valente; amvalente@ciencias.ulisboa.pt

**Keywords**: Ruthenium, palladium, N-methylated PTA, heterobimetallic complex, antiproliferative activity, breast cancer, biological properties

**Contents**

**Synthesis and characterization**…..................………………………………………....3

- [RuCp(DMSO-κ*S*)(HdmoPTA)(PPh_3_)](CF_3_SO_3_)_2_ (**8**)….........................…………….3
- [RuCp(PPh_3_)_2_(dmoPTA-1κ*P*-2κ^2^-*N,N’*-PdCl_2_)](CF_3_SO_3_) (**11**) ………....…...............7

**Single Crystal X-ray diffraction data**………………………...………………………11

**Biological evaluation**……………………......…………...…………….................…...18

- Stability tests in DMSO-d_6_ and DMSO-d_6_/D_2_O under air atmosphere..….......….18
- Cell lines and culture conditions………………….……………....…..…………...23
- Compound cytotoxicity evaluated using the MTT assay…………...………….…23
- Cell death measurement using flow cytometry – the annexin V/PI assay…...….24
- Complex uptake and distribution by ICP-MS…………........................................25
- Binding interaction with Human Serum Albumin by Steady-State and Time-Resolved Fluorescence Emission………………………….…………......….….…25

**Synthesis and characterization**

**[RuCp(DMSO-κ*S*)(HdmoPTA)(PPh_3_)](CF_3_SO_3_)_2_ (8)**

32.6 mg AgCF_3_SO_3_ (0.127 mmol) were added into a solution of 50 mg [RuClCp(HdmoPTA)(PPh_3_)](CF_3_SO_3_) (**7**) (0.0635 mmol) in 0.5 mL of DMSO. A white AgCl precipitate is formed and the mixture is stirred for 24h, filtered with celite and washed with 1 mL of EtOH. 5 mL of Et_2_O is added to the filtered solution and a whitish precipitate appears. It is filtered and washed with Et_2_O (3 x 3 mL) and left to dry under vacuum. Yield: 53.5 mg, 86%. Good enough crystals for X-ray diffraction were obtained by slow evaporation of a DMSO solution of **12**. Solubility: S_25ºC,acetone_ 40.8 mg/cm^3^, S_25ºC,MeOH_ 19.2 mg/cm^3^, S_25ºC,DMSO_ 76.4 mg/cm^3^. C_34_H_43_F_6_N_3_O_7_P_2_RuS_3_ (979.07 g·mol^-1^). Calcd: C 41.67; H 4.26; N 4.29; S 9.79. Found: C 41.27; H 4.52; N 4.47; S 9.51. IR: 1438 (w), 1252 (s), 1226 (m), 1151 (m), 1097 (w), 1033 (s), 997 (m), 934 (m), 756 (m), 704(m), 698 (m), 641 (s). ^1^H RMN (500.13 MHz, acetone-d_6_, 25ºC) δ (ppm): 2.50 + 2.54 (d + d, ^2^*J*_HH_ = 1.46 Hz, ^2^*J*_HH_ = 1.44 Hz, 3H + 3H, *CH_3_*NCH_2_P_HdmoPTA_), 3.01 + 3.67 (s + s, 3H + 3H, (*CH_3_*)_2_SO), 3.42 - 3.66 + 4.30 (m + m, 6H, N*CH_2_*P_HdmoPTA_), 4.02 + 4.51 (d + m, ^2^*J*_HH_ = 11.61 Hz, 4H, N*CH_2_*N_HdmoPTA_), 5.44 (s, 5H, Cp), 7.56 - 7.67 (m, 15H, PPh_3_). ^13^C{^1^H} RMN (125.76 MHz, acetone-d_6_, 25ºC) δ (ppm): 42.31 + 42.45 (d +d, ^3^*J*_PC_ = 5.24 Hz, ^3^*J*_PC_ = 4.93 Hz, *CH_3_*NCH_2_P_HdmoPTA_), 54.23 + 58.98 (s + s, (*CH_3_*)_2_SO), 48.16 + 48.34 + 55.68 + 55.85 + 56.24 + 56.40 (m + m + m + m, N*CH_2_*P_HdmoPTA_), 74.97 + 75.05 (d + d, ^3^*J*_PC_ = 3.74 Hz, ^3^*J*_PC_ = 3.20 Hz, N*CH_2_*N_HdmoPTA_), 85.72 (s, Cp), 129.16 – 133.90 (m, PPh_3_). ^31^P{^1^H} RMN (202.46 MHz, acetone-d_6_, 25ºC) δ (ppm): -5.89 (d, ^2^*J*_PP_ = 37.64 Hz, HdmoPTA), 41.48 (d, ^2^*J*_PP_ = 37.45 Hz, PPh_3_).





**Figure S1**. IR (ATR) of **8**.


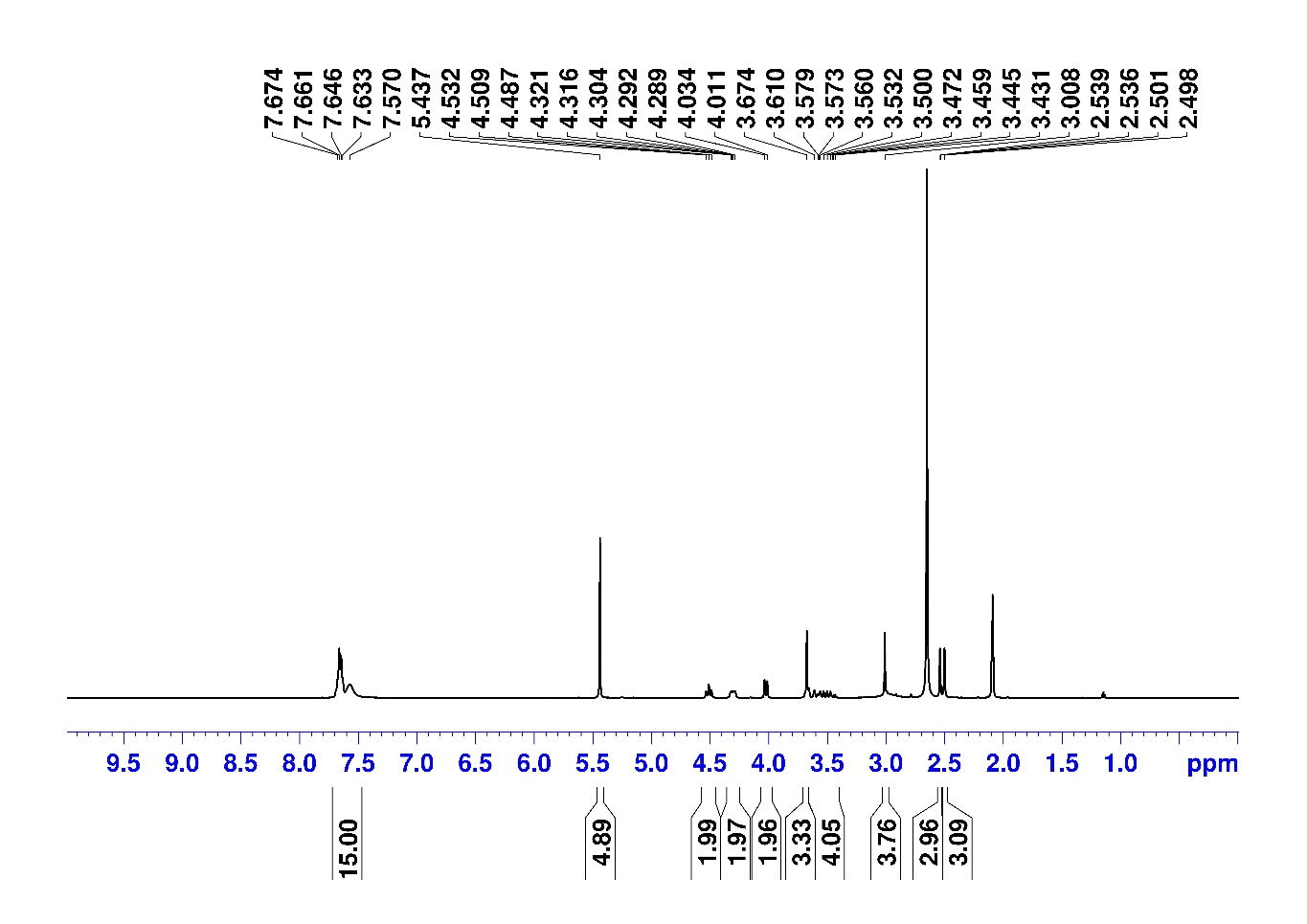


**Figure S2**. ^1^H NMR (500.13 MHz, 25ºC, acetone-d_6_) of **8**.

**
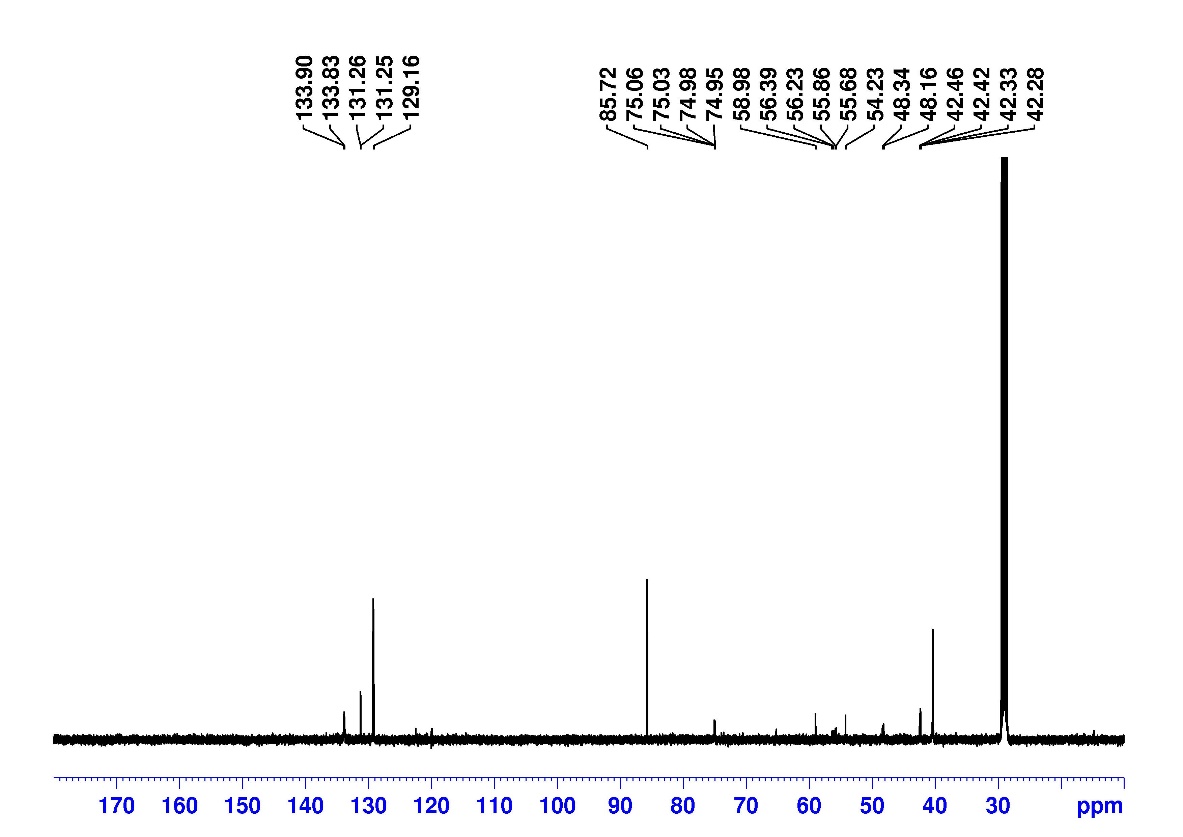
**

**Figure S3**. ^13^C{^1^H} NMR (125.76 MHz, 25ºC, acetone-d_6_) of **8**.

**
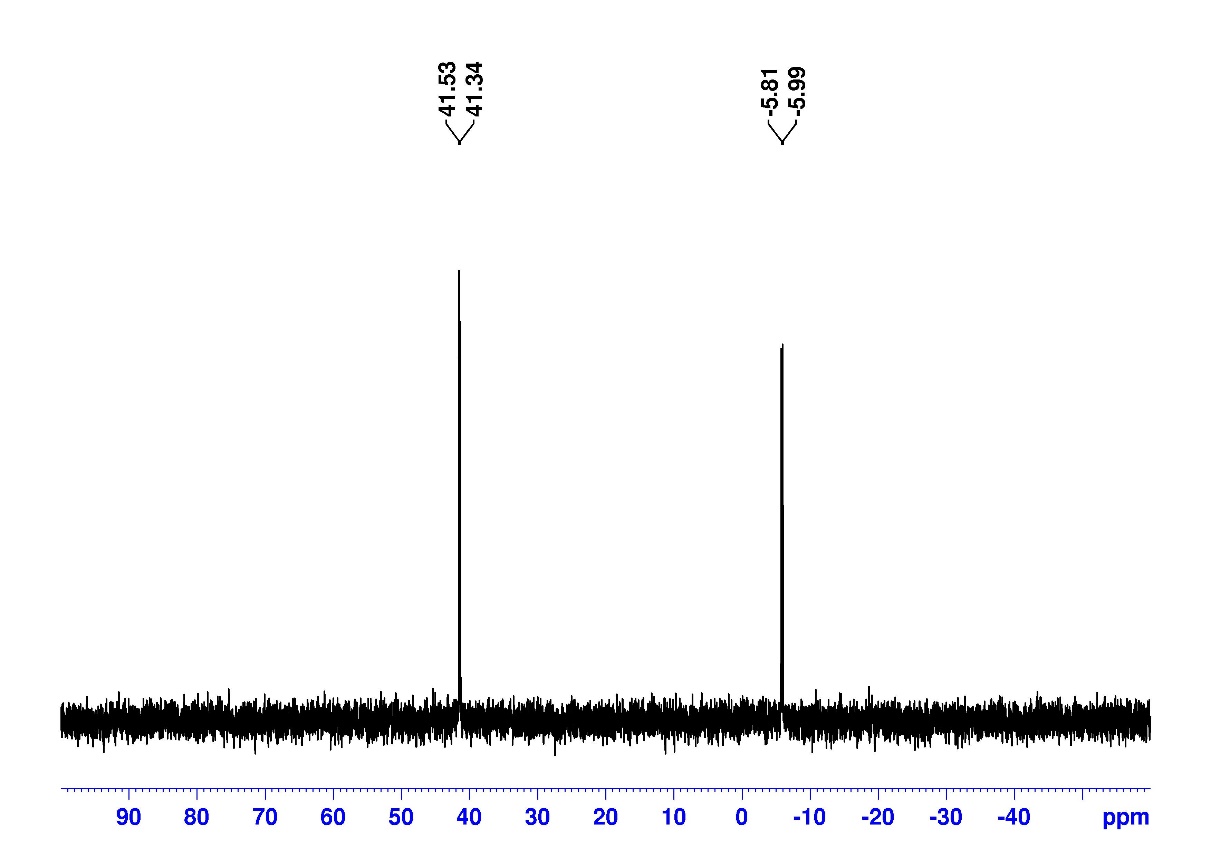
**

**Figure S4**. ^31^P{^1^H} NMR (202.46 MHz, 25ºC, acetone-d_6_) of **8**.


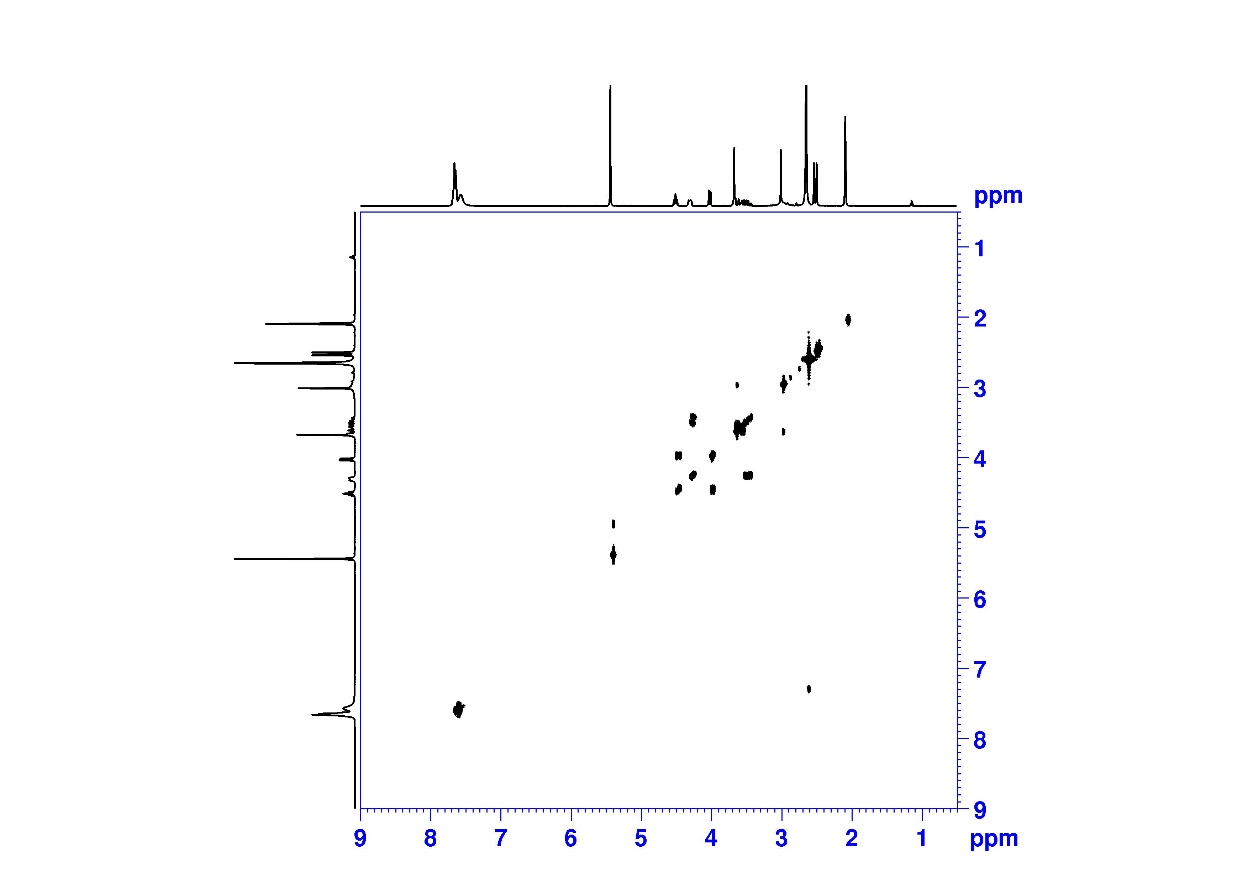


**Figure S5**. ^1^H-^1^H COSY NMR (500.13 MHz, 500.13 MHz, 25ºC, acetone-d_6_) of **8**.

**
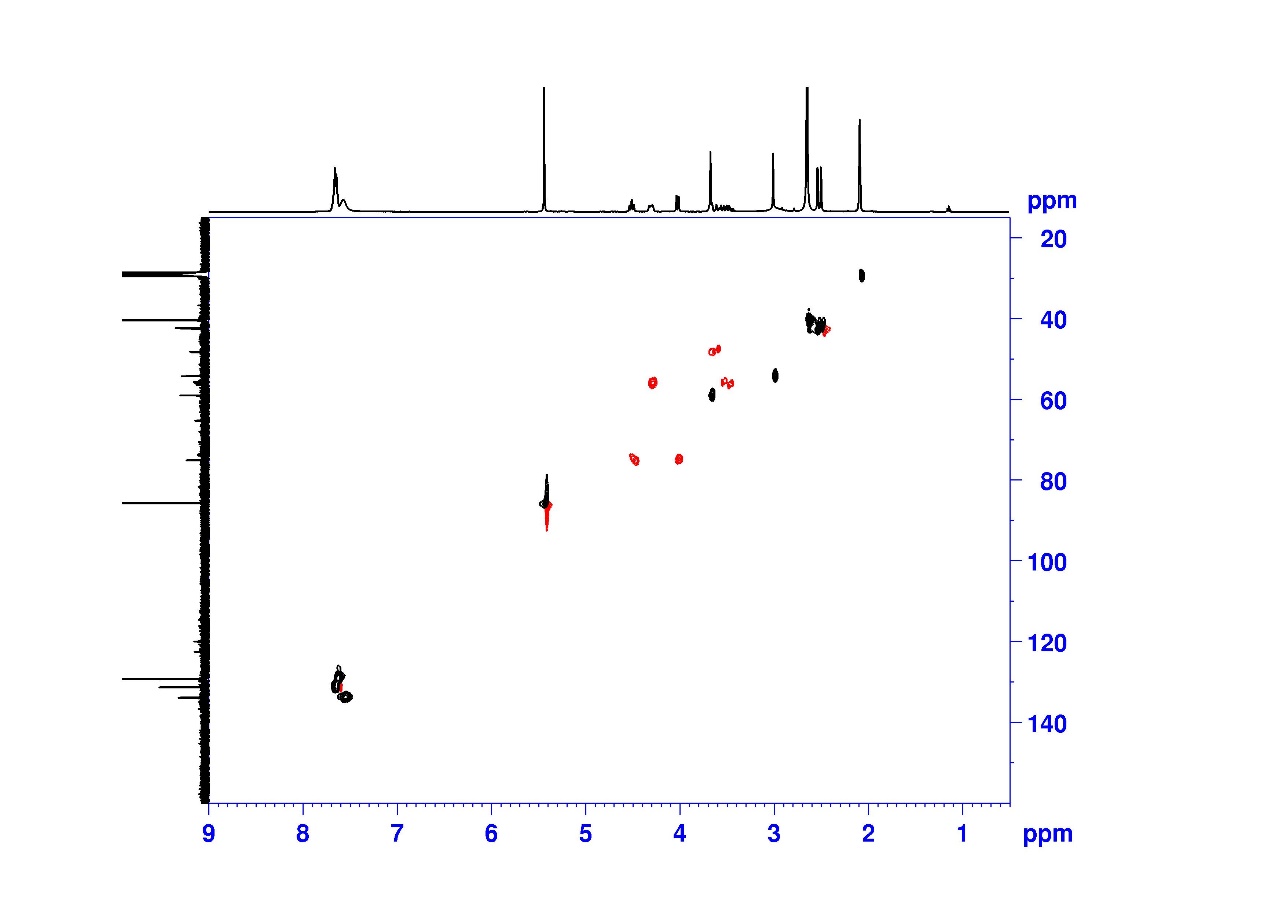
**

**Figure S6**. ^1^H-^13^C HSQC NMR (500.13 MHz, 125.76 MHz, 25ºC, acetone-d_6_) of **8**.

**[RuCp(PPh_3_)_2_(dmoPTA-1κ*P*-2κ^2^-*N,N’*-PdCl_2_)](CF_3_SO_3_) (11)**

(NBu_4_)_2_[PdCl_4_] (68 mg, 0,098 mmol) was added into a solution of [RuCp(PPh_3_)_2_(dmoPTA)](CF_3_SO_3_) (**10**) (100 mg, 0,098 mmol) in dry MeOH (20 mL). The mixture was stirred at room temperature for 30 minutes and the solvent removed. The pale brown residue was dissolved in CHCl_3_ (10 mL) and it is filtered by gravity. The filtered dissolution was concentrated under reduced pressure and Et_2_O was added drop to drop until pale brown precipitate was observed. The resulting precipitate was suction filtered, washed with Et_2_O (3 x 20 mL) and dried under vacuum. Yield: 62.9 mg, 54%. Solubility: S_25ºC,CHCl3_ 4.8 mg/cm^3^, S_25ºC,DMF_ 17 mg/cm^3^, S_25ºC,DMSO_ 25.2 mg/cm^3^. C_49_H_51_Cl_2_F_3_N_3_O_3_P_3_PdRuS (1189,03 g·mol^-1^). Calcd: C 49.45; H 4.32; N 3.53; S 2.69. Found: C 49.64; H 4.47; N 3,39; S 2.48. IR: 2958 (w); 1433 (m); 1275 (s), 1158 (s); 826 (s). ^1^H RMN (600.13 MHz, CDCl_3_, 25ºC) δ (ppm): 2.45 (s, 6H, N*CH_3_*), 3.10 + 4.01 (m + m, 4H, P*CH_2_*NCH_3_), 3.45 (m, 2H, P*CH_2_*N), 4.24 (m, 4H, N*CH_2_*N), 5.16 (s, 5H, Cp), 6.89-7.60 ppm (m, 30H, aromatics, PPh_3_). ^13^C{^1^H} RMN (150.90 MHz, CDCl_3_, 25ºC) δ (ppm): 53.04 (m, N*CH_3_*), 60.27 (m, P*CH_2_*N), 61.77 (m, P*CH_2_*NCH3), 78.25 (m, N*CH_2_*N), 86.50 (s, Cp), 128.45-136.51 (PPh_3_). ^31^P{^1^H} RMN (242.94 MHz, CDCl_3_, 20ºC) δ (ppm): -10.48 (m, dmoPTA-PdCl_2_), 39.51 (d, ^2^*J*_PP_ = 38.92 Hz, PPh_3_).

**

**

**Figure S7.** IR (KBr) of **11.**

**
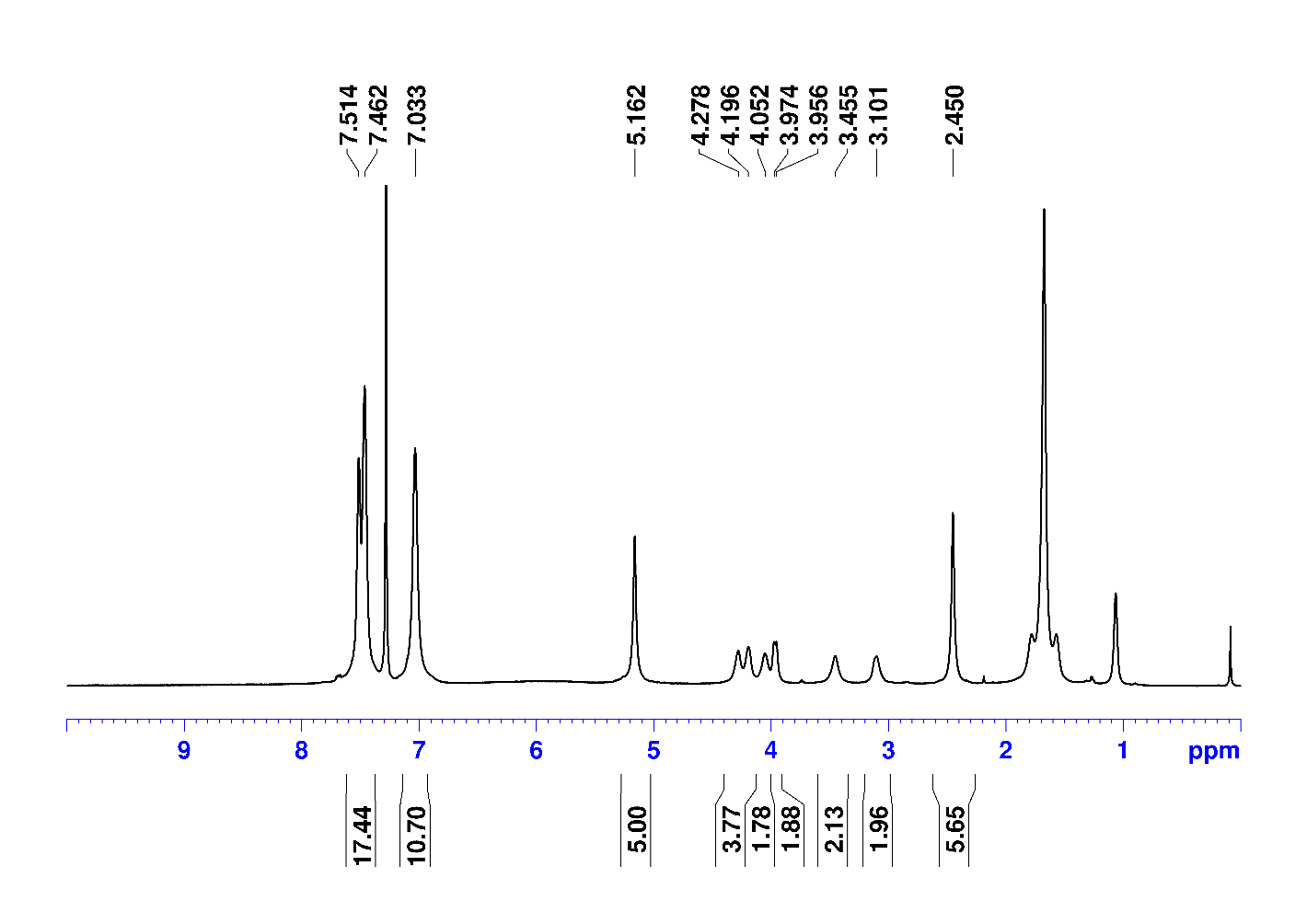
**

**Figure S8.** ^1^H NMR (600.13MHz, CDCl_3_, 25ºC) of **11.**

**
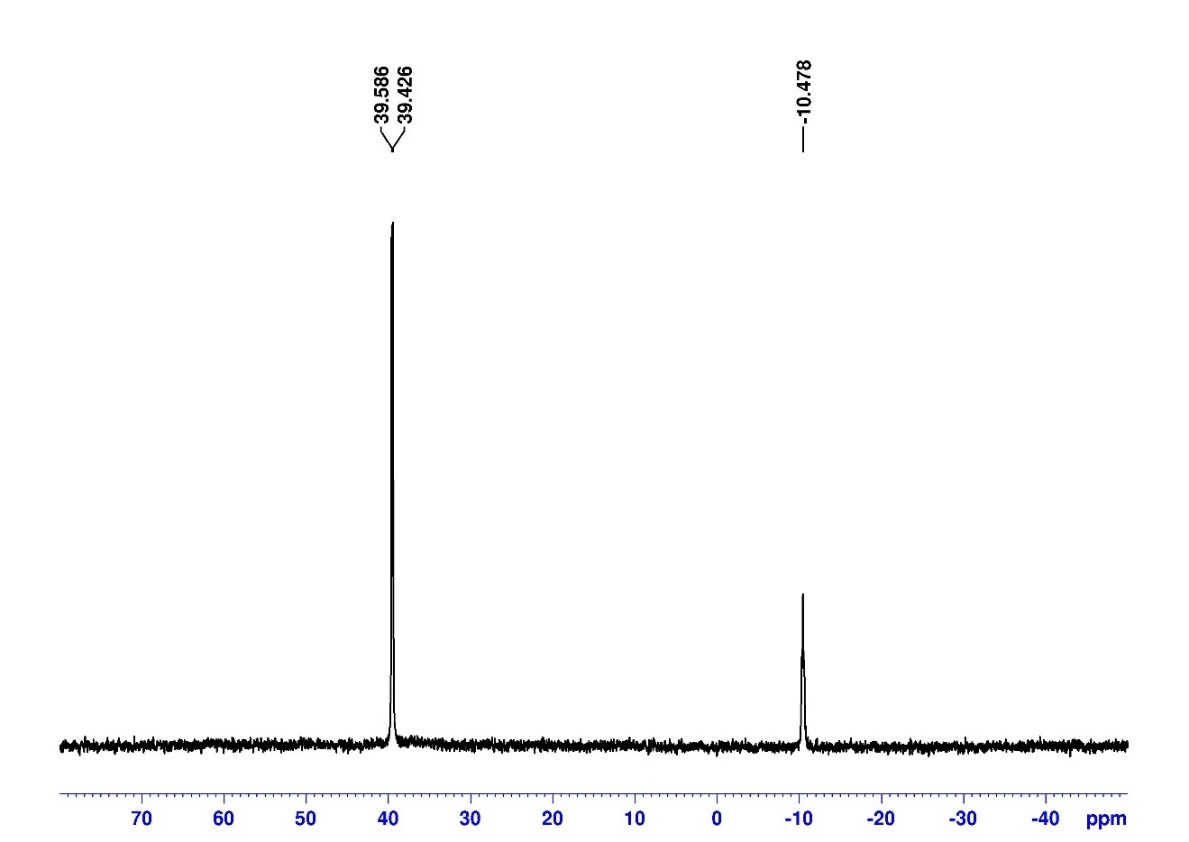
**

**Figure S9.** ^31^P{^1^H} NMR (242.94MHz, CDCl_3_, 25ºC) of **11.**

**
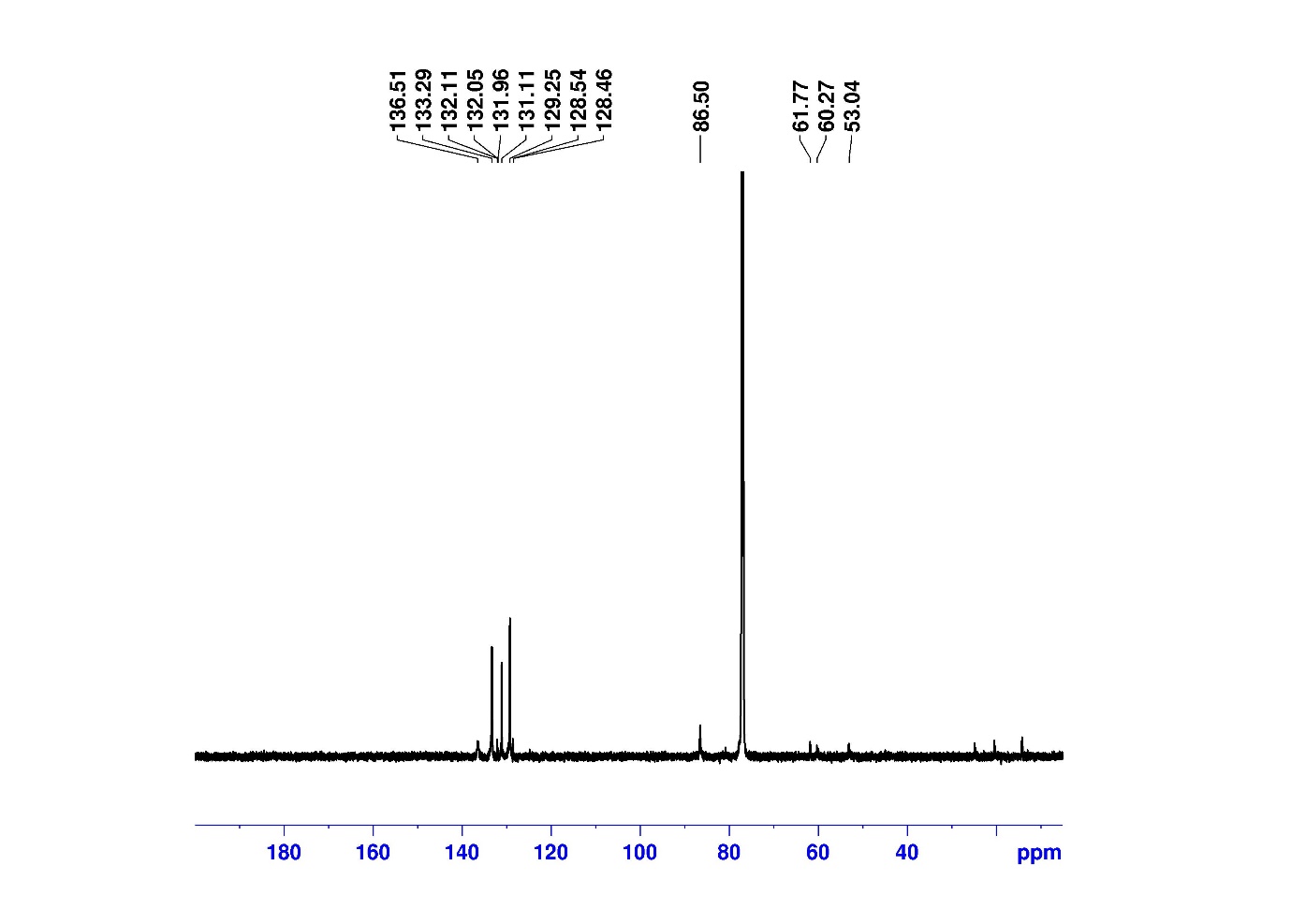
**

**Figure S10.** ^13^C{^1^H} NMR (150.90MHz, CDCl_3_, 25ºC) of **11.**

**
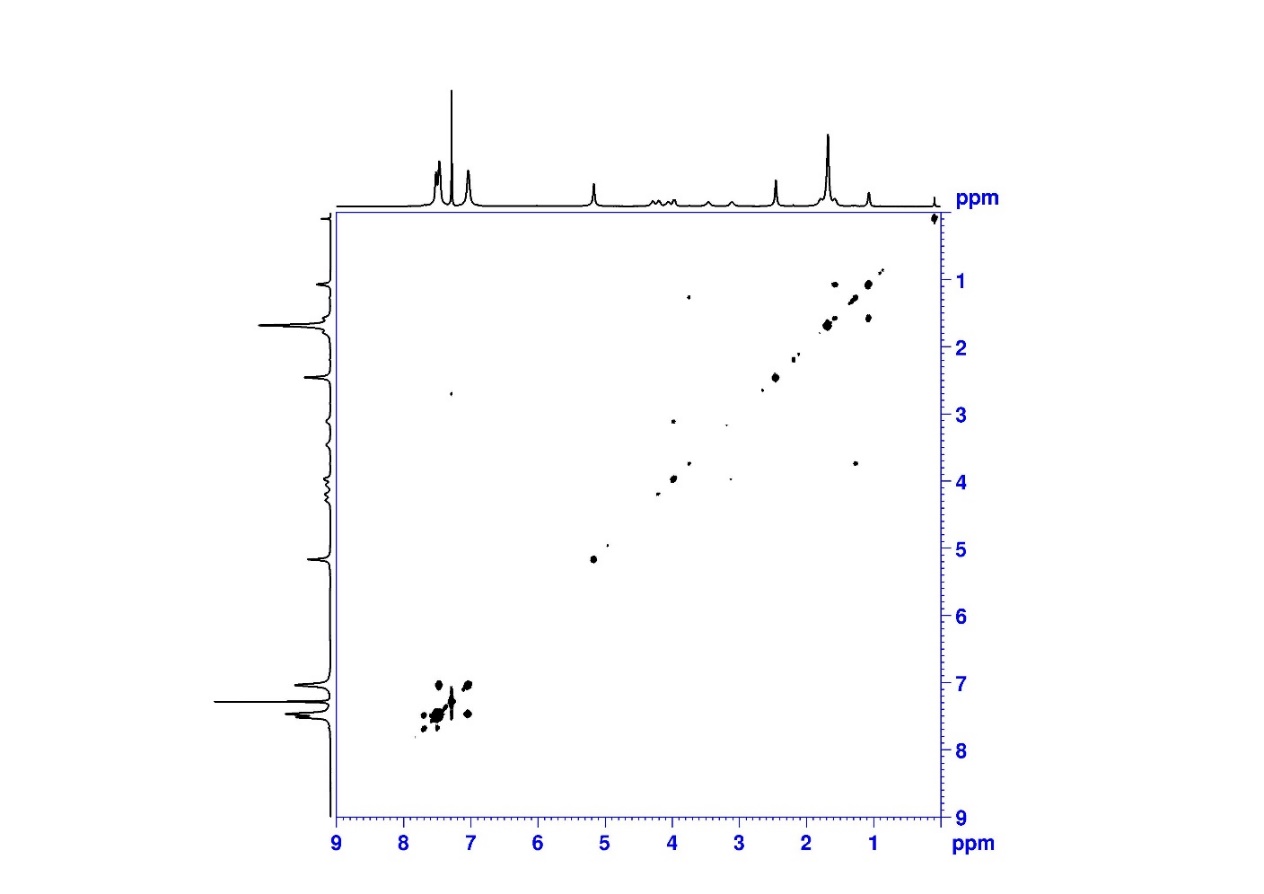
**

**Figure S11**. ^1^H-^1^H COSY NMR (600.13MHz, 600.13MHz, CDCl_3_, 25ºC) of **11.**


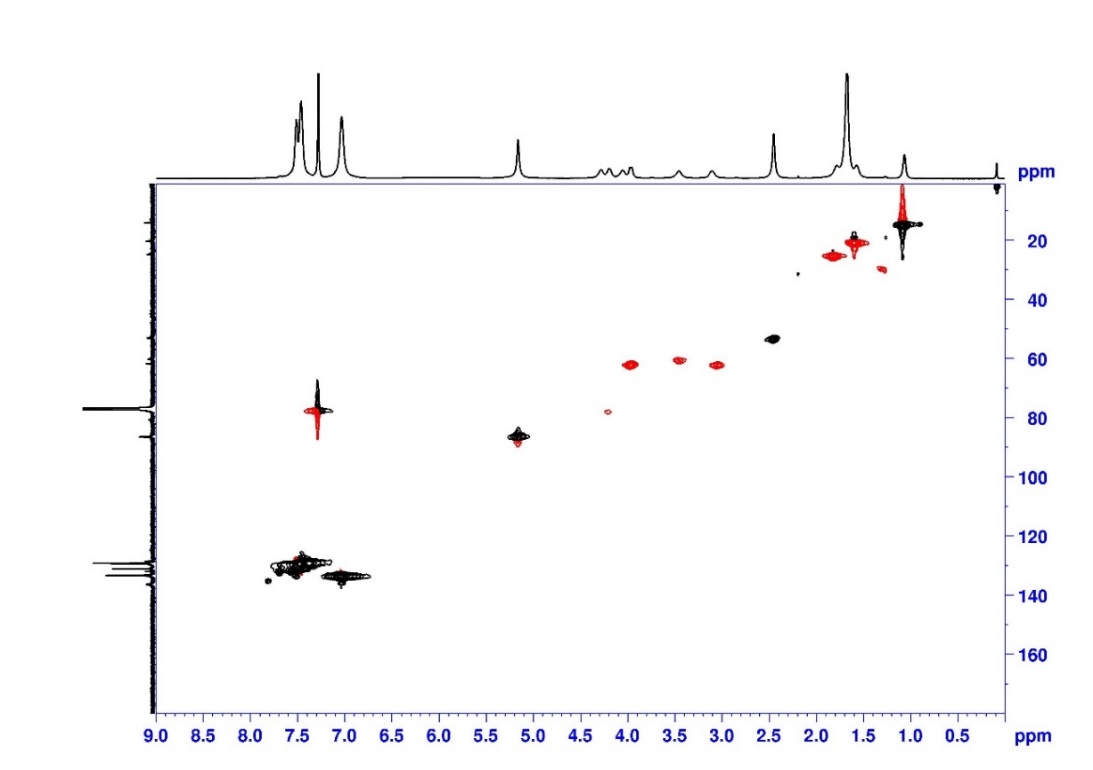


**Figure S12**. ^1^H-^13^C HSQC NMR (600.13MHz, 150.90MHz, CDCl_3_, 25ºC) of **11.**

**Single Crystal X-ray diffraction data**

| **Table S1**. Crystal data and structure refinement for **8** and **11**. | | |  |
| --- | --- | --- | --- |
|  | **8** |  | **11** |
| Empirical formula | C_37_H_49_N_3_P_2_S_3_O_8_F_6_Ru | | C_53_H_55_Cl_14_F_3_N_3_O_3_P_3_RuSPd |
| Formula weight | 1036.98 | | 1667.74 |
| Temperature/K | 301.60 | | 100.0 |
| Crystal system | monoclinic | | triclinic |
| Space group | P2_1_/c | | P-1 |
| a/Å | 9.5483(2) | | 12.8359(7) |
| b/Å | 11.6728(2) | | 14.4650(8) |
| c/Å | 40.0824(8) | | 19.5733(12) |
| α/° | 90 | | 92.140(2) |
| β/° | 95.3710(10) | | 104.143(2) |
| γ/° | 90 | | 111.8940(10) |
| Volume/Å^3^ | 4447.79(15) | | 3236.4(3) |
| Z | 4 | | 2 |
| ρ_calc_g/cm^3^ | 1.549 | | 1.711 |
| μ/mm^‑1^ | 0.642 | | 1.249 |
| F(000) | 2128.0 | | 1668.0 |
| Crystal size/mm^3^ | 0.4 × 0.4 × 0.3 | | 0.3 × 0.1 × 0.1 |
| Radiation | MoKα (λ = 0.71073) | | MoKα (λ = 0.71073) |
| 2Θ range for data collection/° | 4.042 to 63.012 | | 3.972 to 60.068 |
| Index ranges | -14 ≤ h ≤ 14, -17 ≤ k ≤ 17, -58 ≤ l ≤ 58 | | -18 ≤ h ≤ 18, -20 ≤ k ≤ 20, -27 ≤ l ≤ 27 |
| Reflections collected | 121454 | | 152203 |
| Independent reflections | 14781 [R_int_ = 0.0504, R_sigma_ = 0.0293] | | 18933 [R_int_ = 0.0334, R_sigma_ = 0.0196] |
| Data/restraints/parameters | 14781/511/551 | | 18933/7/921 |
| Goodness-of-fit on F^2^ | 1.061 | | 1.145 |
| Final R indexes [I>=2σ (I)] | R_1_ = 0.0622, wR_2_ = 0.1508 | | R_1_ = 0.0360, wR_2_ = 0.0750 |
| Final R indexes [all data] | R_1_ = 0.0800, wR_2_ = 0.1620 | | R_1_ = 0.0386, wR_2_ = 0.0761 |
| Largest diff. peak/hole / e Å^-3^ | 0.88/-1.19 | | 1.36/-0.97 |

| **Table S2**. Bond Lengths for **8**. | | | | | | |
| --- | --- | --- | --- | --- | --- | --- |
| **Atom** | **Atom** | **Length/Å** |  | **Atom** | **Atom** | **Length/Å** |
| Ru1 | P2 | 2.3635(9) |  | C20 | C25 | 1.383(5) |
| Ru1 | P1 | 2.3107(8) |  | C13 | C8 | 1.398(5) |
| Ru1 | S1 | 2.2764(9) |  | C13 | C12 | 1.391(6) |
| Ru1 | C29 | 2.226(4) |  | C8 | C9 | 1.389(5) |
| Ru1 | C27 | 2.239(4) |  | F4TA | C2T | 1.278(10) |
| Ru1 | C28 | 2.236(4) |  | C29 | C28 | 1.411(6) |
| Ru1 | C30 | 2.229(4) |  | C29 | C30 | 1.412(7) |
| Ru1 | C26 | 2.231(4) |  | C21 | C22 | 1.384(5) |
| P2 | C14 | 1.833(3) |  | C27 | C28 | 1.391(6) |
| P2 | C20 | 1.833(3) |  | C27 | C26 | 1.403(6) |
| P2 | C8 | 1.839(3) |  | F1T | C1T | 1.329(8) |
| P1 | C3 | 1.851(3) |  | C22 | C23 | 1.384(7) |
| P1 | C2 | 1.841(3) |  | F6TA | C2T | 1.301(9) |
| P1 | C1 | 1.847(4) |  | C12 | C11 | 1.372(8) |
| S1 | O1 | 1.472(3) |  | C19 | C18 | 1.385(6) |
| S1 | C32 | 1.790(4) |  | C9 | C10 | 1.385(6) |
| S1 | C31 | 1.792(4) |  | C25 | C24 | 1.390(6) |
| S2TA | O4TA | 1.416(9) |  | C17 | C18 | 1.369(7) |
| S2TA | O6TA | 1.438(9) |  | C17 | C16 | 1.361(8) |
| S2TA | C2T | 1.790(8) |  | F2T | C1T | 1.334(9) |
| S2TA | O5TA | 1.410(7) |  | O1A | C2A | 1.197(7) |
| S1T | O3T | 1.438(4) |  | C15 | C16 | 1.390(6) |
| S1T | O2T | 1.420(4) |  | F3T | C1T | 1.338(8) |
| S1T | O1T | 1.407(4) |  | C30 | C26 | 1.390(7) |
| S1T | C1T | 1.771(8) |  | C11 | C10 | 1.367(7) |
| N2 | C2 | 1.461(4) |  | C2A | C1A | 1.456(8) |
| N2 | C5 | 1.425(5) |  | C2A | C3A | 1.474(8) |
| N2 | C4 | 1.454(5) |  | C23 | C24 | 1.366(7) |
| N3 | C3 | 1.501(4) |  | F5TA | C2T | 1.448(10) |
| N3 | C5 | 1.520(5) |  | C2T | S2TB | 1.756(14) |
| N3 | C7 | 1.486(5) |  | C2T | F4TB | 1.27(2) |
| N1 | C4 | 1.484(5) |  | C2T | F6TB | 1.217(19) |
| N1 | C1 | 1.473(4) |  | C2T | F5TB | 1.692(18) |
| N1 | C6 | 1.476(5) |  | S2TB | O5TB | 1.408(19) |
| C14 | C19 | 1.397(5) |  | S2TB | O4TB | 1.47(3) |
| C14 | C15 | 1.378(5) |  | S2TB | O6TB | 1.48(2) |
| C20 | C21 | 1.401(5) |  |  |  |  |

| **Table S3**. Bond Lengths for **11**. | | | | | | |
| --- | --- | --- | --- | --- | --- | --- |
| **Atom** | **Atom** | **Length/Å** |  | **Atom** | **Atom** | **Length/Å** |
| Pd1 | Cl2 | 2.3059(6) |  | C8 | C9 | 1.384(3) |
| Pd1 | Cl1 | 2.2876(6) |  | C26 | C27 | 1.396(3) |
| Pd1 | N1 | 2.1092(18) |  | C26 | C31 | 1.389(3) |
| Pd1 | N2 | 2.1058(19) |  | C32 | C37 | 1.397(3) |
| Ru1 | P1 | 2.3077(5) |  | C32 | C33 | 1.400(3) |
| Ru1 | P3 | 2.3650(5) |  | C47 | C46 | 1.419(3) |
| Ru1 | P2 | 2.3717(6) |  | C36 | C37 | 1.386(3) |
| Ru1 | C45 | 2.229(2) |  | C36 | C35 | 1.388(3) |
| Ru1 | C48 | 2.241(2) |  | C43 | C38 | 1.391(3) |
| Ru1 | C44 | 2.239(2) |  | C43 | C42 | 1.390(3) |
| Ru1 | C47 | 2.248(2) |  | C13 | C12 | 1.386(4) |
| Ru1 | C46 | 2.239(2) |  | C38 | C39 | 1.400(3) |
| P1 | C2 | 1.841(2) |  | C27 | C28 | 1.388(3) |
| P1 | C1 | 1.844(2) |  | C19 | C18 | 1.391(3) |
| P1 | C3 | 1.843(2) |  | C31 | C30 | 1.393(3) |
| P3 | C26 | 1.832(2) |  | C39 | C40 | 1.385(3) |
| P3 | C32 | 1.837(2) |  | C20 | C25 | 1.402(3) |
| P3 | C38 | 1.825(2) |  | C20 | C21 | 1.391(3) |
| P2 | C14 | 1.847(2) |  | C33 | C34 | 1.391(3) |
| P2 | C8 | 1.840(2) |  | C9 | C10 | 1.394(3) |
| P2 | C20 | 1.843(2) |  | C15 | C16 | 1.392(3) |
| S1T | O2TB | 1.489(19) |  | C25 | C24 | 1.389(3) |
| S1T | O1TB | 1.477(16) |  | C35 | C34 | 1.380(4) |
| S1T | O3TB | 1.414(7) |  | C40 | C41 | 1.388(4) |
| S1T | C1TA | 1.887(10) |  | C41 | C42 | 1.386(4) |
| S1T | C1TB | 1.726(10) |  | C12 | C11 | 1.379(4) |
| S1T | O2TA | 1.452(6) |  | C11 | C10 | 1.380(4) |
| S1T | O1TA | 1.38(2) |  | C28 | C29 | 1.367(4) |
| S1T | O3TA | 1.37(2) |  | C24 | C23 | 1.390(5) |
| Cl23 | C1C | 1.752(3) |  | C18 | C17 | 1.381(3) |
| Cl10 | C2C | 1.692(7) |  | C21 | C22 | 1.393(3) |
| Cl22 | C1C | 1.766(3) |  | C17 | C16 | 1.381(3) |
| Cl24 | C1C | 1.740(3) |  | C30 | C29 | 1.390(4) |
| Cl16 | C3CA | 1.758(5) |  | C22 | C23 | 1.379(5) |
| Cl17 | C3CA | 1.748(5) |  | F2TA | C1TA | 1.285(10) |
| Cl12 | C2C | 1.822(5) |  | C2C | Cl15 | 1.560(12) |
| Cl11 | C2C | 1.616(6) |  | C2C | Cl14 | 1.930(8) |
| Cl18 | C3CA | 1.764(6) |  | C2C | Cl13 | 1.850(8) |
| N1 | C1 | 1.501(3) |  | C1TA | F3TA | 1.326(13) |
| N1 | C4 | 1.507(3) |  | C1TA | F1TA | 1.30(3) |
| N1 | C6 | 1.498(3) |  | Cl19 | C3CB | 1.745(13) |
| N2 | C2 | 1.490(3) |  | Cl20 | C3CB | 1.752(17) |
| N2 | C5 | 1.505(3) |  | Cl21 | C3CB | 1.78(2) |
| N2 | C7 | 1.489(3) |  | F2TB | C1TB | 1.329(11) |
| N3 | C5 | 1.430(3) |  | C1TB | F1TB | 1.339(11) |
| N3 | C3 | 1.464(3) |  | Cl3C | C1CA | 1.722(6) |
| N3 | C4 | 1.435(3) |  | Cl1C | C1CA | 1.807(6) |
| F3TB | C1TB | 1.35(2) |  | Cl2C | C1CA | 1.781(7) |
| C14 | C19 | 1.397(3) |  | Cl5C | C1CB | 1.729(10) |
| C14 | C15 | 1.396(3) |  | Cl4C | C1CB | 1.725(10) |
| C45 | C44 | 1.416(3) |  | Cl6C | C1CB | 1.774(10) |
| C45 | C46 | 1.423(3) |  | Cl9C | C1CC | 1.754(10) |
| C48 | C44 | 1.428(3) |  | Cl7C | C1CC | 1.754(10) |
| C48 | C47 | 1.420(3) |  | C1CC | Cl8C | 1.781(10) |
| C8 | C13 | 1.405(3) |  |  |  |  |

| **Table S4**. Bond Angles for **8**. | | | | | | | | |
| --- | --- | --- | --- | --- | --- | --- | --- | --- |
| **Atom** | **Atom** | **Atom** | **Angle/˚** |  | **Atom** | **Atom** | **Atom** | **Angle/˚** |
| P1 | Ru1 | P2 | 95.00(3) |  | C15 | C14 | P2 | 123.4(3) |
| S1 | Ru1 | P2 | 94.95(3) |  | C15 | C14 | C19 | 119.1(3) |
| S1 | Ru1 | P1 | 93.80(3) |  | C21 | C20 | P2 | 119.0(3) |
| C29 | Ru1 | P2 | 112.38(14) |  | C25 | C20 | P2 | 122.2(3) |
| C29 | Ru1 | P1 | 95.24(12) |  | C25 | C20 | C21 | 118.7(3) |
| C29 | Ru1 | S1 | 150.24(13) |  | C12 | C13 | C8 | 120.2(4) |
| C29 | Ru1 | C27 | 61.26(16) |  | N2 | C2 | P1 | 110.2(2) |
| C29 | Ru1 | C28 | 36.88(16) |  | C13 | C8 | P2 | 121.9(3) |
| C29 | Ru1 | C30 | 36.96(19) |  | C9 | C8 | P2 | 119.9(3) |
| C29 | Ru1 | C26 | 60.97(18) |  | C9 | C8 | C13 | 118.2(3) |
| C27 | Ru1 | P2 | 103.30(12) |  | N2 | C5 | N3 | 113.3(3) |
| C27 | Ru1 | P1 | 154.32(12) |  | N2 | C4 | N1 | 111.5(3) |
| C27 | Ru1 | S1 | 102.27(11) |  | N1 | C1 | P1 | 113.5(3) |
| C28 | Ru1 | P2 | 89.98(12) |  | C28 | C29 | Ru1 | 72.0(2) |
| C28 | Ru1 | P1 | 127.69(12) |  | C28 | C29 | C30 | 107.3(4) |
| C28 | Ru1 | S1 | 137.64(11) |  | C30 | C29 | Ru1 | 71.6(3) |
| C28 | Ru1 | C27 | 36.23(15) |  | C22 | C21 | C20 | 120.3(4) |
| C30 | Ru1 | P2 | 148.83(14) |  | C28 | C27 | Ru1 | 71.8(2) |
| C30 | Ru1 | P1 | 94.03(13) |  | C28 | C27 | C26 | 107.5(4) |
| C30 | Ru1 | S1 | 114.14(15) |  | C26 | C27 | Ru1 | 71.4(2) |
| C30 | Ru1 | C27 | 61.19(17) |  | C23 | C22 | C21 | 120.3(4) |
| C30 | Ru1 | C28 | 61.24(17) |  | C11 | C12 | C13 | 120.3(5) |
| C30 | Ru1 | C26 | 36.31(18) |  | C29 | C28 | Ru1 | 71.2(2) |
| C26 | Ru1 | P2 | 139.44(13) |  | C27 | C28 | Ru1 | 72.0(2) |
| C26 | Ru1 | P1 | 124.69(13) |  | C27 | C28 | C29 | 108.5(4) |
| C26 | Ru1 | S1 | 90.76(13) |  | C18 | C19 | C14 | 120.1(4) |
| C26 | Ru1 | C27 | 36.57(16) |  | C10 | C9 | C8 | 120.8(4) |
| C26 | Ru1 | C28 | 60.57(16) |  | C20 | C25 | C24 | 120.1(4) |
| C14 | P2 | Ru1 | 112.64(13) |  | C16 | C17 | C18 | 120.2(4) |
| C14 | P2 | C20 | 103.20(16) |  | C17 | C18 | C19 | 120.0(4) |
| C14 | P2 | C8 | 103.00(16) |  | C14 | C15 | C16 | 119.7(4) |
| C20 | P2 | Ru1 | 113.04(12) |  | C29 | C30 | Ru1 | 71.4(2) |
| C20 | P2 | C8 | 101.00(16) |  | C26 | C30 | Ru1 | 71.9(3) |
| C8 | P2 | Ru1 | 121.77(11) |  | C26 | C30 | C29 | 107.6(4) |
| C3 | P1 | Ru1 | 120.77(11) |  | C10 | C11 | C12 | 120.1(4) |
| C2 | P1 | Ru1 | 122.01(11) |  | C27 | C26 | Ru1 | 72.0(2) |
| C2 | P1 | C3 | 97.79(16) |  | C30 | C26 | Ru1 | 71.8(2) |
| C2 | P1 | C1 | 97.00(17) |  | C30 | C26 | C27 | 109.1(4) |
| C1 | P1 | Ru1 | 113.85(12) |  | O1A | C2A | C1A | 121.5(6) |
| C1 | P1 | C3 | 100.99(16) |  | O1A | C2A | C3A | 120.5(6) |
| O1 | S1 | Ru1 | 121.59(12) |  | C1A | C2A | C3A | 117.9(6) |
| O1 | S1 | C32 | 105.8(2) |  | C24 | C23 | C22 | 119.5(4) |
| O1 | S1 | C31 | 105.1(2) |  | C23 | C24 | C25 | 121.1(4) |
| C32 | S1 | Ru1 | 112.12(16) |  | C11 | C10 | C9 | 120.3(4) |
| C32 | S1 | C31 | 98.1(2) |  | C17 | C16 | C15 | 120.9(5) |
| C31 | S1 | Ru1 | 111.40(17) |  | F4TA | C2T | S2TA | 112.6(6) |
| O4TA | S2TA | O6TA | 114.4(5) |  | F4TA | C2T | F6TA | 113.5(8) |
| O4TA | S2TA | C2T | 108.8(5) |  | F4TA | C2T | F5TA | 106.0(6) |
| O6TA | S2TA | C2T | 103.8(5) |  | F6TA | C2T | S2TA | 112.7(5) |
| O5TA | S2TA | O4TA | 114.5(5) |  | F6TA | C2T | F5TA | 104.8(7) |
| O5TA | S2TA | O6TA | 114.4(5) |  | F5TA | C2T | S2TA | 106.4(5) |
| O5TA | S2TA | C2T | 98.9(5) |  | F4TB | C2T | S2TB | 121.1(10) |
| O3T | S1T | C1T | 102.8(3) |  | F4TB | C2T | F5TB | 100.5(12) |
| O2T | S1T | O3T | 114.7(3) |  | F6TB | C2T | S2TB | 125.5(10) |
| O2T | S1T | C1T | 103.7(3) |  | F6TB | C2T | F4TB | 108.3(13) |
| O1T | S1T | O3T | 114.5(3) |  | F6TB | C2T | F5TB | 94.8(11) |
| O1T | S1T | O2T | 116.2(3) |  | F5TB | C2T | S2TB | 97.4(7) |
| O1T | S1T | C1T | 102.3(4) |  | F1T | C1T | S1T | 114.0(6) |
| C5 | N2 | C2 | 114.2(3) |  | F1T | C1T | F2T | 105.7(6) |
| C5 | N2 | C4 | 113.7(3) |  | F1T | C1T | F3T | 104.7(7) |
| C4 | N2 | C2 | 113.0(3) |  | F2T | C1T | S1T | 113.0(6) |
| C3 | N3 | C5 | 112.3(3) |  | F2T | C1T | F3T | 105.9(7) |
| C7 | N3 | C3 | 110.8(3) |  | F3T | C1T | S1T | 112.8(5) |
| C7 | N3 | C5 | 111.5(3) |  | O5TB | S2TB | C2T | 92.5(9) |
| C1 | N1 | C4 | 111.9(3) |  | O5TB | S2TB | O4TB | 104.3(12) |
| C1 | N1 | C6 | 110.1(3) |  | O5TB | S2TB | O6TB | 107.2(13) |
| C6 | N1 | C4 | 111.9(3) |  | O4TB | S2TB | C2T | 113.4(11) |
| N3 | C3 | P1 | 114.3(2) |  | O4TB | S2TB | O6TB | 130.3(14) |
| C19 | C14 | P2 | 117.4(3) |  | O6TB | S2TB | C2T | 102.6(11) |

| **Table S5**. Bond Angles for **11**. | | | | | | | | |
| --- | --- | --- | --- | --- | --- | --- | --- | --- |
| **Atom** | **Atom** | **Atom** | **Angle/˚** |  | **Atom** | **Atom** | **Atom** | **Angle/˚** |
| Cl1 | Pd1 | Cl2 | 85.25(2) |  | C9 | C8 | C13 | 118.3(2) |
| N1 | Pd1 | Cl2 | 175.82(5) |  | N3 | C5 | N2 | 115.02(18) |
| N1 | Pd1 | Cl1 | 93.68(5) |  | C27 | C26 | P3 | 121.97(18) |
| N2 | Pd1 | Cl2 | 92.96(5) |  | C31 | C26 | P3 | 119.46(17) |
| N2 | Pd1 | Cl1 | 178.20(5) |  | C31 | C26 | C27 | 118.5(2) |
| N2 | Pd1 | N1 | 88.12(7) |  | C37 | C32 | P3 | 119.67(16) |
| P1 | Ru1 | P3 | 95.378(19) |  | C37 | C32 | C33 | 118.4(2) |
| P1 | Ru1 | P2 | 97.864(19) |  | C33 | C32 | P3 | 121.85(17) |
| P3 | Ru1 | P2 | 101.30(2) |  | N3 | C3 | P1 | 109.34(15) |
| C45 | Ru1 | P1 | 122.70(6) |  | C45 | C44 | Ru1 | 71.17(12) |
| C45 | Ru1 | P3 | 140.46(6) |  | C45 | C44 | C48 | 108.07(19) |
| C45 | Ru1 | P2 | 84.93(6) |  | C48 | C44 | Ru1 | 71.49(12) |
| C45 | Ru1 | C48 | 61.97(8) |  | C48 | C47 | Ru1 | 71.27(12) |
| C45 | Ru1 | C44 | 36.94(8) |  | C46 | C47 | Ru1 | 71.22(12) |
| C45 | Ru1 | C47 | 61.54(8) |  | C46 | C47 | C48 | 108.7(2) |
| C45 | Ru1 | C46 | 37.14(8) |  | C45 | C46 | Ru1 | 71.05(11) |
| C48 | Ru1 | P1 | 93.94(6) |  | C47 | C46 | Ru1 | 71.90(11) |
| C48 | Ru1 | P3 | 109.28(6) |  | C47 | C46 | C45 | 107.40(19) |
| C48 | Ru1 | P2 | 145.93(6) |  | C37 | C36 | C35 | 119.9(2) |
| C48 | Ru1 | C47 | 36.88(8) |  | C42 | C43 | C38 | 120.2(2) |
| C44 | Ru1 | P1 | 91.95(6) |  | C36 | C37 | C32 | 121.1(2) |
| C44 | Ru1 | P3 | 146.22(6) |  | N3 | C4 | N1 | 113.96(17) |
| C44 | Ru1 | P2 | 110.30(6) |  | C12 | C13 | C8 | 120.5(2) |
| C44 | Ru1 | C48 | 37.17(8) |  | C43 | C38 | P3 | 122.98(16) |
| C44 | Ru1 | C47 | 61.51(8) |  | C43 | C38 | C39 | 118.93(19) |
| C44 | Ru1 | C46 | 61.88(8) |  | C39 | C38 | P3 | 118.08(16) |
| C47 | Ru1 | P1 | 126.92(6) |  | C28 | C27 | C26 | 120.8(2) |
| C47 | Ru1 | P3 | 88.23(6) |  | C18 | C19 | C14 | 120.9(2) |
| C47 | Ru1 | P2 | 133.32(6) |  | C26 | C31 | C30 | 120.3(2) |
| C46 | Ru1 | P1 | 153.17(6) |  | C40 | C39 | C38 | 120.6(2) |
| C46 | Ru1 | P3 | 103.48(6) |  | C25 | C20 | P2 | 120.31(18) |
| C46 | Ru1 | P2 | 96.96(6) |  | C21 | C20 | P2 | 121.06(18) |
| C46 | Ru1 | C48 | 62.01(8) |  | C21 | C20 | C25 | 118.6(2) |
| C46 | Ru1 | C47 | 36.87(8) |  | C34 | C33 | C32 | 120.2(2) |
| C2 | P1 | Ru1 | 119.75(7) |  | C8 | C9 | C10 | 120.9(2) |
| C2 | P1 | C1 | 104.34(9) |  | C16 | C15 | C14 | 120.5(2) |
| C2 | P1 | C3 | 96.59(10) |  | C24 | C25 | C20 | 120.7(3) |
| C1 | P1 | Ru1 | 119.66(7) |  | C34 | C35 | C36 | 119.8(2) |
| C3 | P1 | Ru1 | 116.29(7) |  | C39 | C40 | C41 | 120.0(2) |
| C3 | P1 | C1 | 95.40(10) |  | C42 | C41 | C40 | 119.7(2) |
| C26 | P3 | Ru1 | 120.18(7) |  | C11 | C12 | C13 | 120.4(2) |
| C26 | P3 | C32 | 100.30(10) |  | C12 | C11 | C10 | 119.9(2) |
| C32 | P3 | Ru1 | 112.97(7) |  | C41 | C42 | C43 | 120.4(2) |
| C38 | P3 | Ru1 | 116.59(7) |  | C29 | C28 | C27 | 120.4(3) |
| C38 | P3 | C26 | 103.21(10) |  | C25 | C24 | C23 | 119.9(3) |
| C38 | P3 | C32 | 100.73(10) |  | C17 | C18 | C19 | 120.4(2) |
| C14 | P2 | Ru1 | 111.67(7) |  | C20 | C21 | C22 | 120.4(3) |
| C8 | P2 | Ru1 | 115.25(7) |  | C16 | C17 | C18 | 119.4(2) |
| C8 | P2 | C14 | 99.77(9) |  | C29 | C30 | C31 | 120.2(3) |
| C8 | P2 | C20 | 100.51(11) |  | C23 | C22 | C21 | 120.5(3) |
| C20 | P2 | Ru1 | 124.48(7) |  | C17 | C16 | C15 | 120.7(2) |
| C20 | P2 | C14 | 101.65(10) |  | C35 | C34 | C33 | 120.6(2) |
| O2TB | S1T | C1TB | 104.6(9) |  | C11 | C10 | C9 | 120.0(3) |
| O1TB | S1T | O2TB | 110.7(8) |  | Cl23 | C1C | Cl22 | 109.53(14) |
| O1TB | S1T | C1TB | 104.4(8) |  | Cl24 | C1C | Cl23 | 111.20(17) |
| O3TB | S1T | O2TB | 118.2(8) |  | Cl24 | C1C | Cl22 | 109.56(16) |
| O3TB | S1T | O1TB | 108.9(8) |  | C22 | C23 | C24 | 119.8(3) |
| O3TB | S1T | C1TB | 109.1(5) |  | C28 | C29 | C30 | 119.7(2) |
| O2TA | S1T | C1TA | 100.8(4) |  | Cl16 | C3CA | Cl18 | 111.2(3) |
| O1TA | S1T | C1TA | 101.2(9) |  | Cl17 | C3CA | Cl16 | 109.9(3) |
| O1TA | S1T | O2TA | 119.9(8) |  | Cl17 | C3CA | Cl18 | 109.4(3) |
| O3TA | S1T | C1TA | 105.0(10) |  | Cl10 | C2C | Cl12 | 109.9(3) |
| O3TA | S1T | O2TA | 112.6(8) |  | Cl11 | C2C | Cl10 | 116.7(4) |
| O3TA | S1T | O1TA | 114.3(11) |  | Cl11 | C2C | Cl12 | 112.8(4) |
| C1 | N1 | Pd1 | 108.93(13) |  | Cl15 | C2C | Cl14 | 108.4(4) |
| C1 | N1 | C4 | 110.76(17) |  | Cl15 | C2C | Cl13 | 114.9(4) |
| C4 | N1 | Pd1 | 111.10(13) |  | Cl13 | C2C | Cl14 | 101.1(4) |
| C6 | N1 | Pd1 | 114.03(14) |  | F2TA | C1TA | S1T | 111.2(6) |
| C6 | N1 | C1 | 106.11(16) |  | F2TA | C1TA | F3TA | 109.0(11) |
| C6 | N1 | C4 | 105.78(18) |  | F2TA | C1TA | F1TA | 108.9(10) |
| C2 | N2 | Pd1 | 108.24(13) |  | F3TA | C1TA | S1T | 111.3(7) |
| C2 | N2 | C5 | 110.90(17) |  | F1TA | C1TA | S1T | 111.8(12) |
| C5 | N2 | Pd1 | 110.88(13) |  | F1TA | C1TA | F3TA | 104.3(14) |
| C7 | N2 | Pd1 | 114.20(14) |  | Cl19 | C3CB | Cl20 | 111.6(9) |
| C7 | N2 | C2 | 106.67(16) |  | Cl19 | C3CB | Cl21 | 107.8(9) |
| C7 | N2 | C5 | 105.88(17) |  | Cl20 | C3CB | Cl21 | 109.4(9) |
| C5 | N3 | C3 | 113.94(18) |  | F3TB | C1TB | S1T | 111.4(11) |
| C5 | N3 | C4 | 115.91(19) |  | F2TB | C1TB | S1T | 112.6(7) |
| C4 | N3 | C3 | 114.20(18) |  | F2TB | C1TB | F3TB | 109.8(11) |
| C19 | C14 | P2 | 118.93(16) |  | F2TB | C1TB | F1TB | 105.9(8) |
| C15 | C14 | P2 | 122.92(16) |  | F1TB | C1TB | S1T | 108.2(7) |
| C15 | C14 | C19 | 118.15(19) |  | F1TB | C1TB | F3TB | 108.8(13) |
| N2 | C2 | P1 | 117.63(14) |  | Cl3C | C1CA | Cl1C | 110.6(4) |
| N1 | C1 | P1 | 117.24(15) |  | Cl3C | C1CA | Cl2C | 109.9(4) |
| C44 | C45 | Ru1 | 71.89(12) |  | Cl2C | C1CA | Cl1C | 110.6(3) |
| C44 | C45 | C46 | 108.40(19) |  | Cl5C | C1CB | Cl6C | 106.4(5) |
| C46 | C45 | Ru1 | 71.81(12) |  | Cl4C | C1CB | Cl5C | 110.7(6) |
| C44 | C48 | Ru1 | 71.34(12) |  | Cl4C | C1CB | Cl6C | 114.1(5) |
| C47 | C48 | Ru1 | 71.85(12) |  | Cl9C | C1CC | Cl8C | 109.5(7) |
| C47 | C48 | C44 | 107.37(19) |  | Cl7C | C1CC | Cl9C | 110.7(8) |
| C13 | C8 | P2 | 120.74(17) |  | Cl7C | C1CC | Cl8C | 107.1(7) |
| C9 | C8 | P2 | 120.67(16) |  |  |  |  |  |

| **Table S6.** Selected bond lengths and angles for **8** and **11**. | | | | | | | | | | | | |  |
| --- | --- | --- | --- | --- | --- | --- | --- | --- | --- | --- | --- | --- | --- |
| **Bond Angles / º** | | | | | | | | | | | | |  |
| **8** | | | | | | **11** | | | | | | |  |
| P1 | Ru1 | | P2 | 95.00(3) | | P1 | | Ru1 | | P3 | 95.378(19) | |  |
| S1 | Ru1 | | P2 | 94.95(3) | | P1 | | Ru1 | | P2 | 97.864(19) | |  |
| S1 | Ru1 | | P1 | 93.80(3) | | P3 | | Ru1 | | P2 | 101.30(2) | |  |
| O1 | S1 | | Ru1 | 121.59(12) | | Cl1 | | Pd1 | | Cl2 | 85.25(2) | |  |
| O1 | S1 | | C32 | 105.8(2) | | N1 | | Pd1 | | Cl2 | 175.82(5) | |  |
| O1 | S1 | | C31 | 105.1(2) | | N1 | | Pd1 | | Cl1 | 93.68(5) | |  |
| C32 | S1 | | Ru1 | 112.12(16) | | N2 | | Pd1 | | Cl2 | 92.96(5) | |  |
| C32 | S1 | | C31 | 98.1(2) | | N2 | | Pd1 | | Cl1 | 178.20(5) | |  |
| C31 | S1 | | Ru1 | 111.40(17) | | N2 | | Pd1 | | N1 | 88.12(7) | |  |
| **Bond Lengths / Å** | | | | | | | | | | | | |  |
| **8** | | | | | | | **11** | | | | | |  |
| Ru1 | | P2 | | | 2.3635(9) | | Ru1 | | P1 | | | 2.3077(5) |  |
| Ru1 | | P1 | | | 2.3107(8) | | Ru1 | | P3 | | | 2.3650(5) |  |
| Ru1 | | S1 | | | 2.2764(9) | | Ru1 | | P2 | | | 2.3717(6) |  |
| Ru1 | | C29 | | | 2.226(4) | | Ru1 | | C45 | | | 2.229(2) |  |
| Ru1 | | C27 | | | 2.239(4) | | Ru1 | | C48 | | | 2.241(2) |  |
| Ru1 | | C28 | | | 2.236(4) | | Ru1 | | C44 | | | 2.239(2) |  |
| Ru1 | | C30 | | | 2.229(4) | | Ru1 | | C47 | | | 2.248(2) |  |
| Ru1 | | C26 | | | 2.231(4) | | Ru1 | | C46 | | | 2.239(2) |  |
| S1 | | O1 | | | 1.472(3) | | Pd1 | | Cl2 | | | 2.3059(6) |  |
| S1 | | C32 | | | 1.790(4) | | Pd1 | | Cl1 | | | 2.2876(6) |  |
| S1 | | C31 | | | 1.792(4) | | Pd1 | | N1 | | | 2.1092(18) |  |
|  | |  | | |  | | Pd1 | | N2 | | | 2.1058(19) |  |

**Biological evaluation**

**Stability tests in DMSO and DMSO/D_2_O under air atmosphere**

All compounds were studied for their stability in 100% DMSO-d_6_ and DMSO-d_6_/D_2_O mixture by using NMR spectroscopy in common aerobic conditions. All experiments were performed by a similar procedure: the complex (0.01 g) was introduced into a 5 mm NMR tube and dissolved in 0.5 mL of degassed solvent (DMSO-d_6_ and a 1:1 mixture of DMSO-d_6_/D_2_O). The solution was left at room temperature and monitored by ^31^P{^1^H} NMR first every 15 minutes, and later in longer time periods. Stability was also monitored at incubation cell-based assays temperature (37ºC).


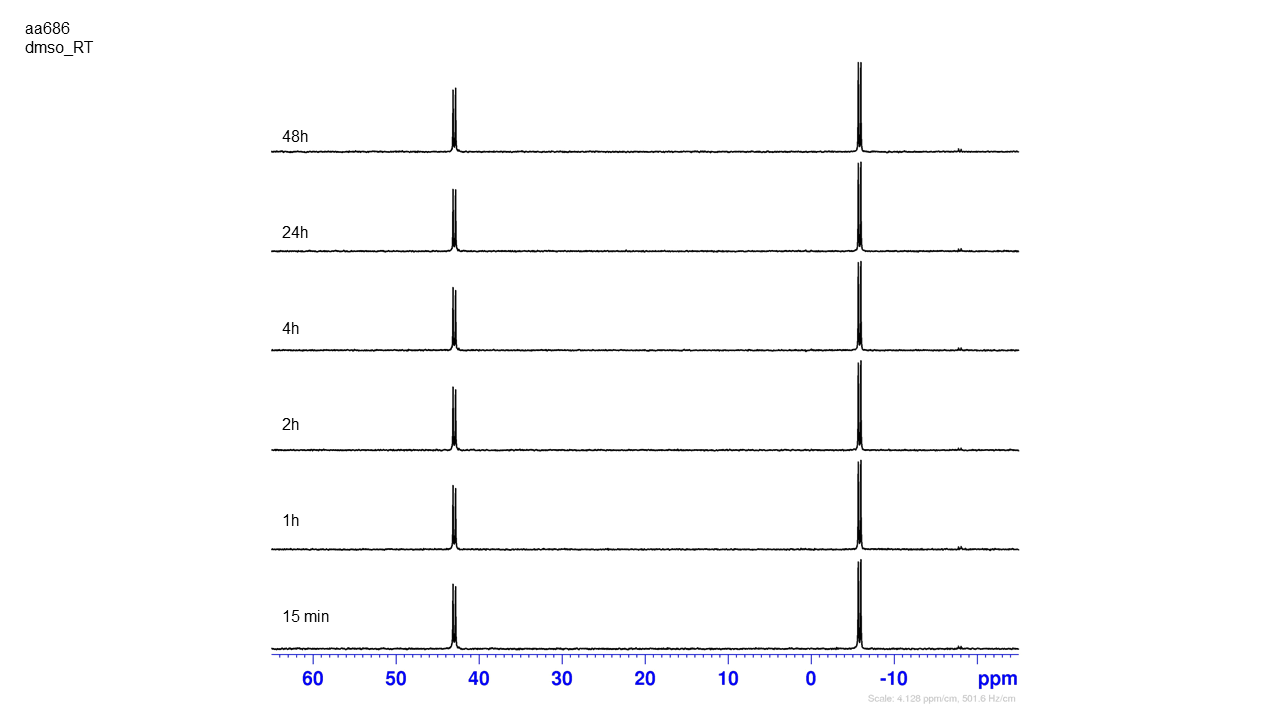


**Figure S13**. ^31^P{^1^H} NMR (121.49 MHz, DMSO-d_6_, 25ºC) of **8** vs. time

**
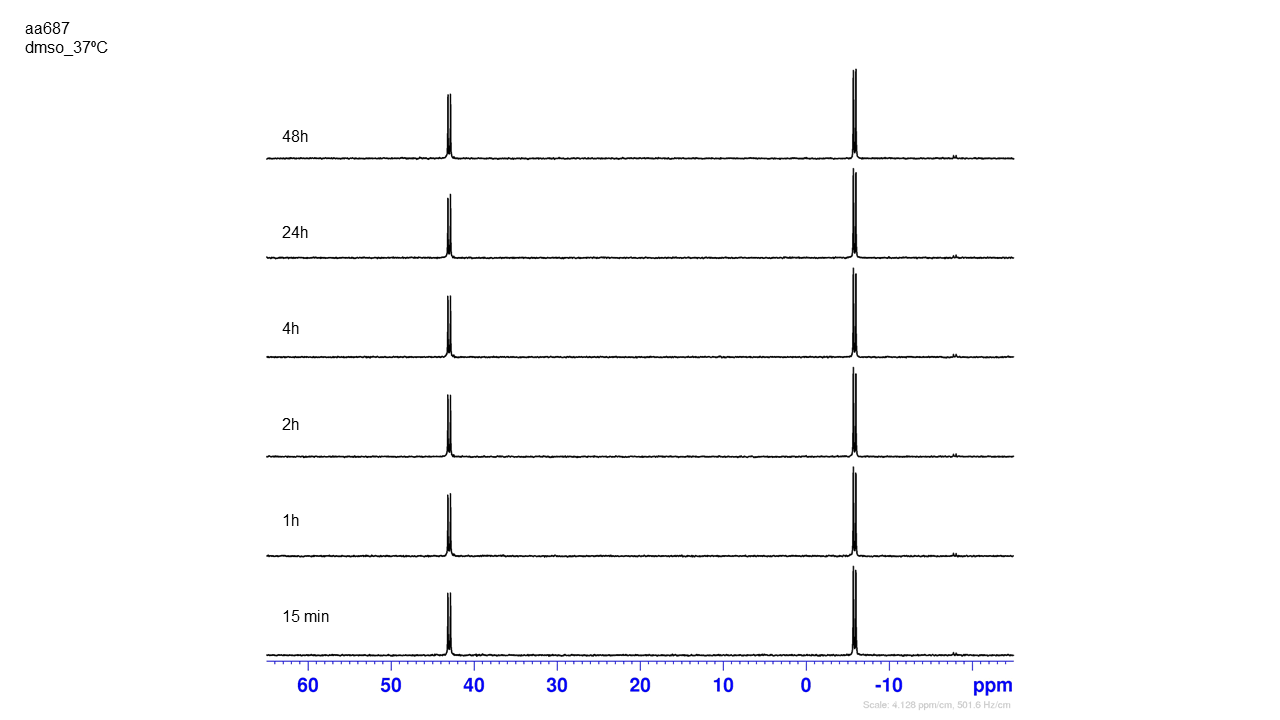
**

**Figure S14**. ^31^P{^1^H} NMR (121.49 MHz, DMSO-d_6_, 37ºC) of **8** vs. time

**
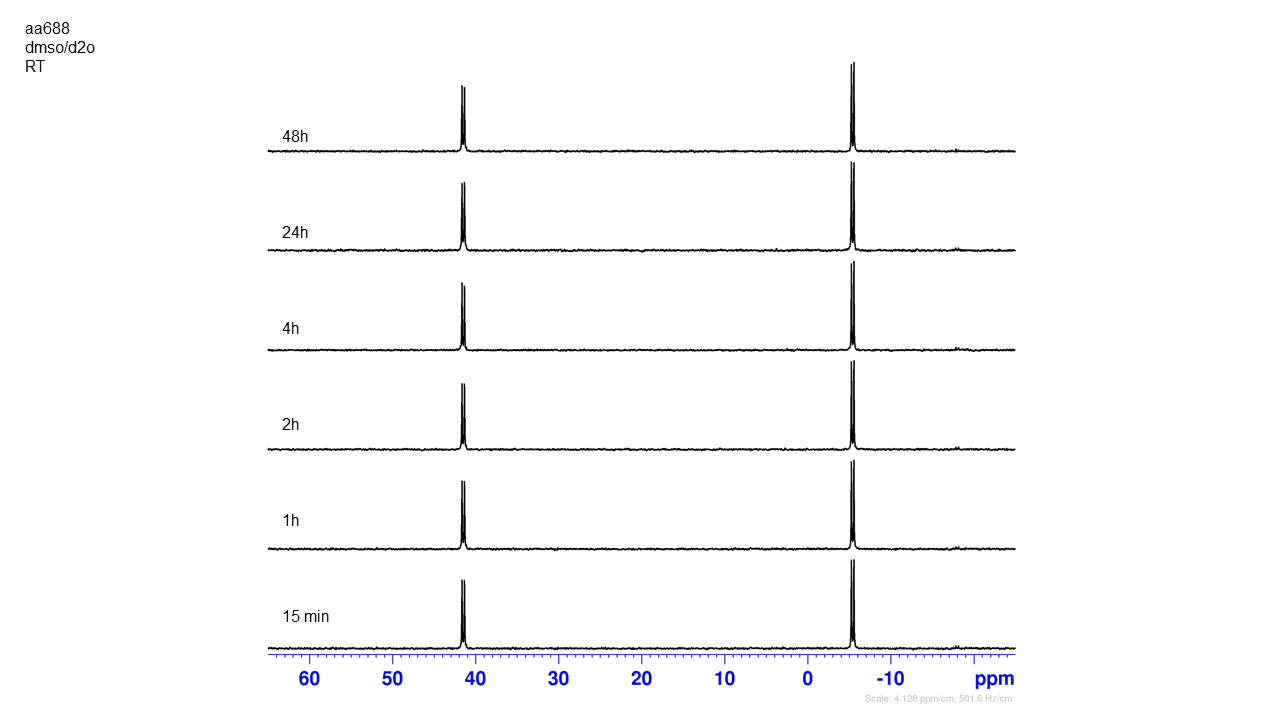
**

**Figure S15**. ^31^P{^1^H} NMR (121.49 MHz, DMSO-d_6_/D_2_O, 25ºC) of **8** vs. time

**
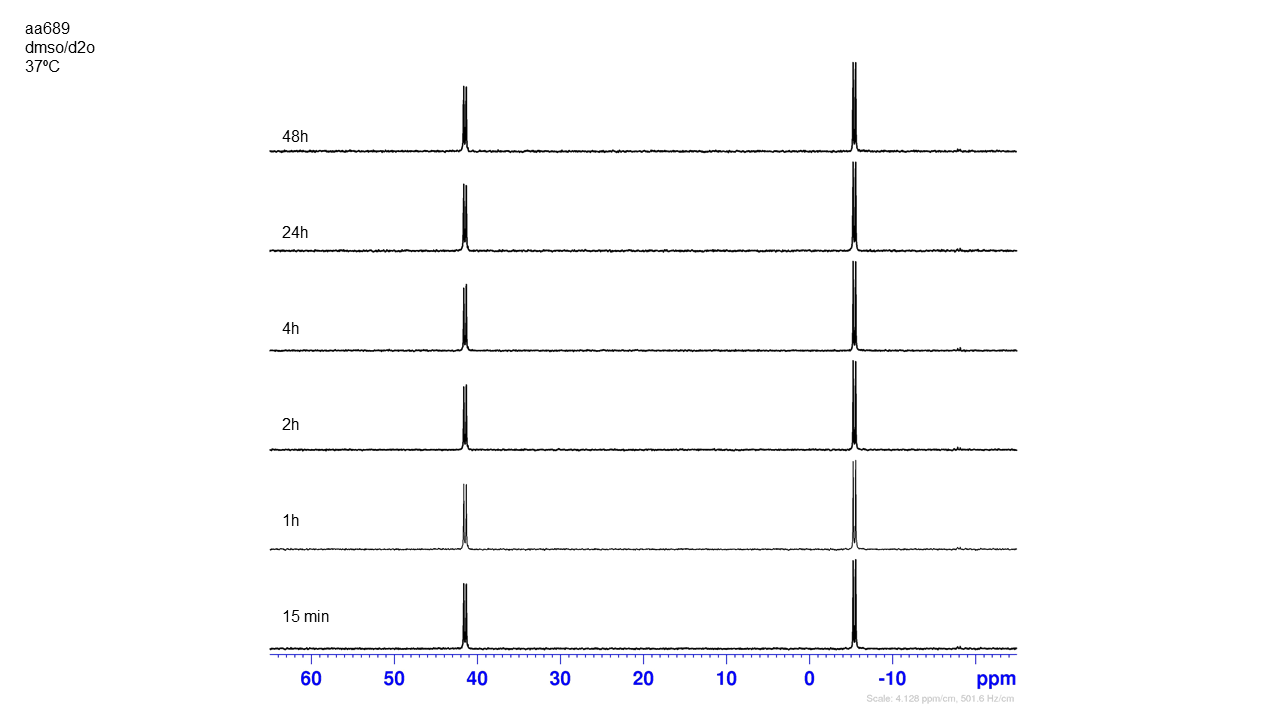
**

**Figure S16**. ^31^P{^1^H} NMR (121.49 MHz, DMSO-d_6_/D_2_O, 37ºC) of **8** vs. time

**
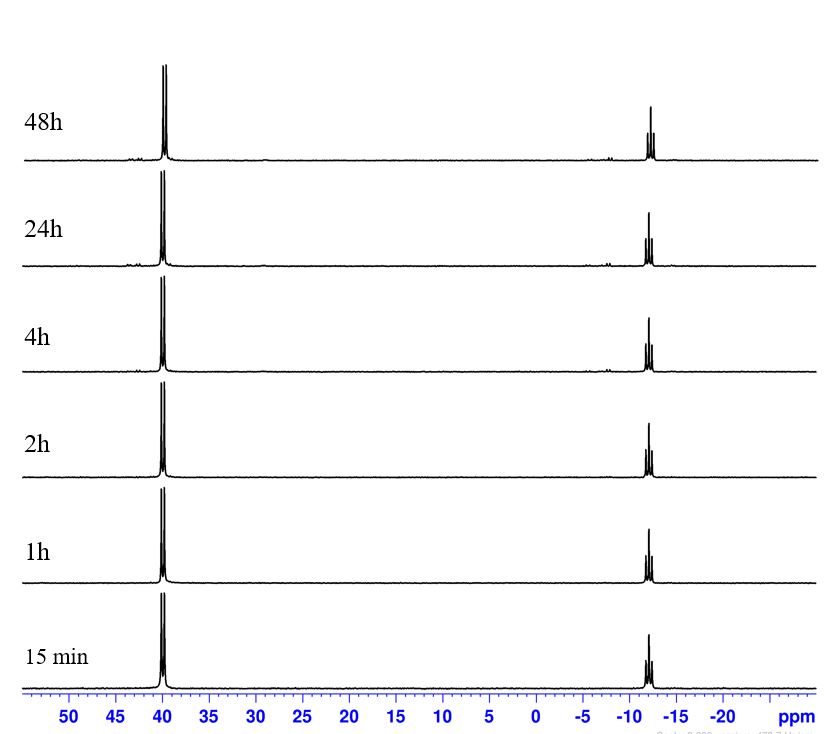
**

**Figure S17**. ^31^P{^1^H} NMR (121.49 MHz, DMSO-d_6_, 25ºC) of **11** vs. time


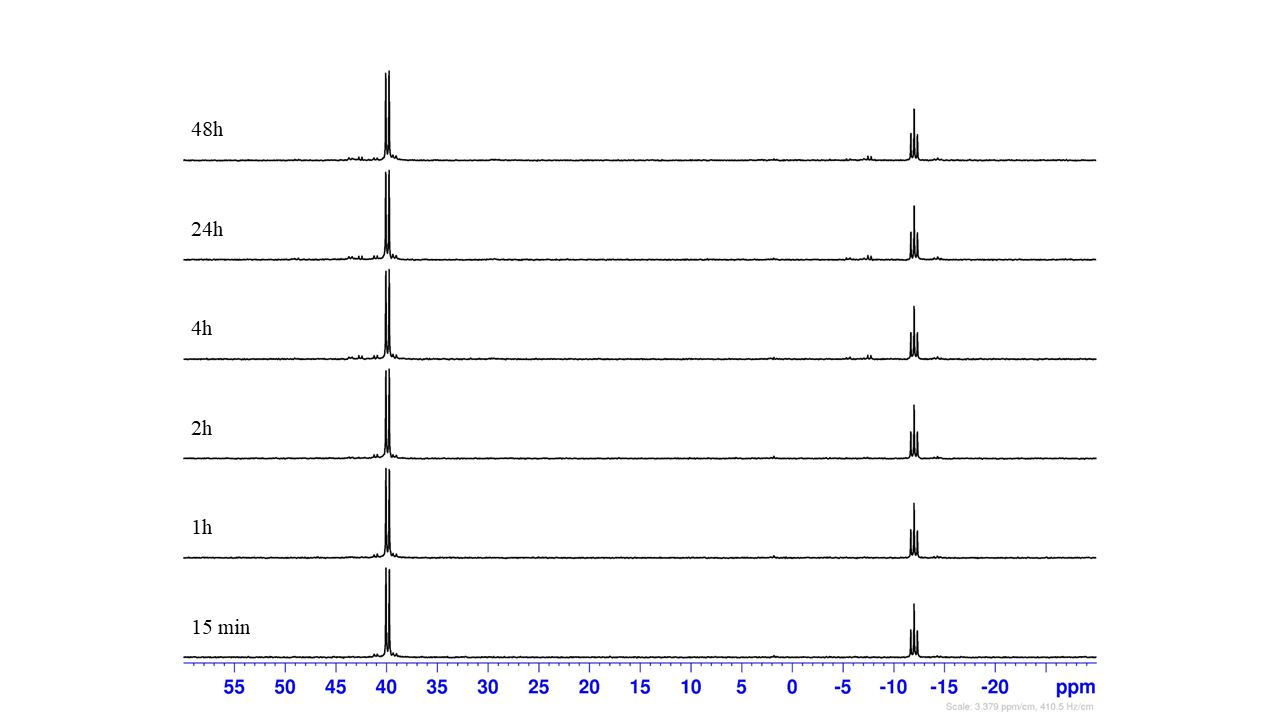


**Figure S18**. ^31^P{^1^H} NMR (121.49 MHz, DMSO-d_6_, 37ºC) of **11** vs. time

**
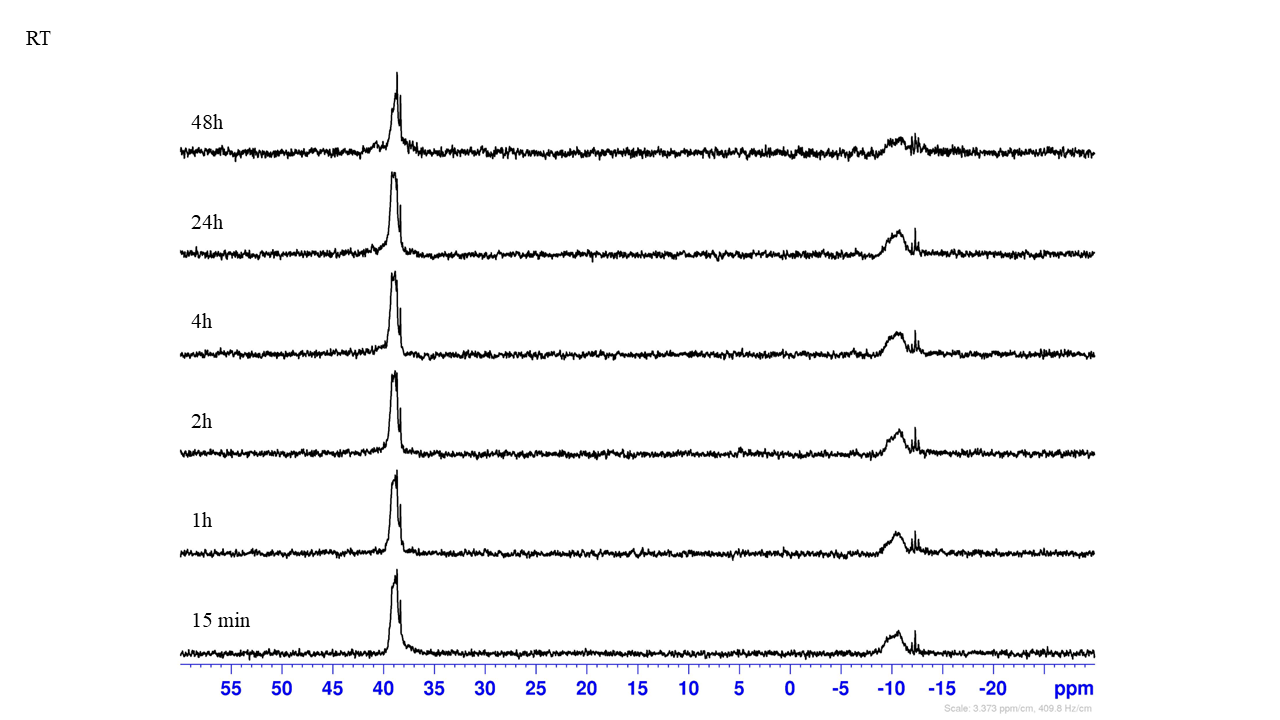
**

**Figure S19**. ^31^P{^1^H} NMR (121.49 MHz, DMSO-d_6_/D_2_O, 25ºC) of **11** vs. time


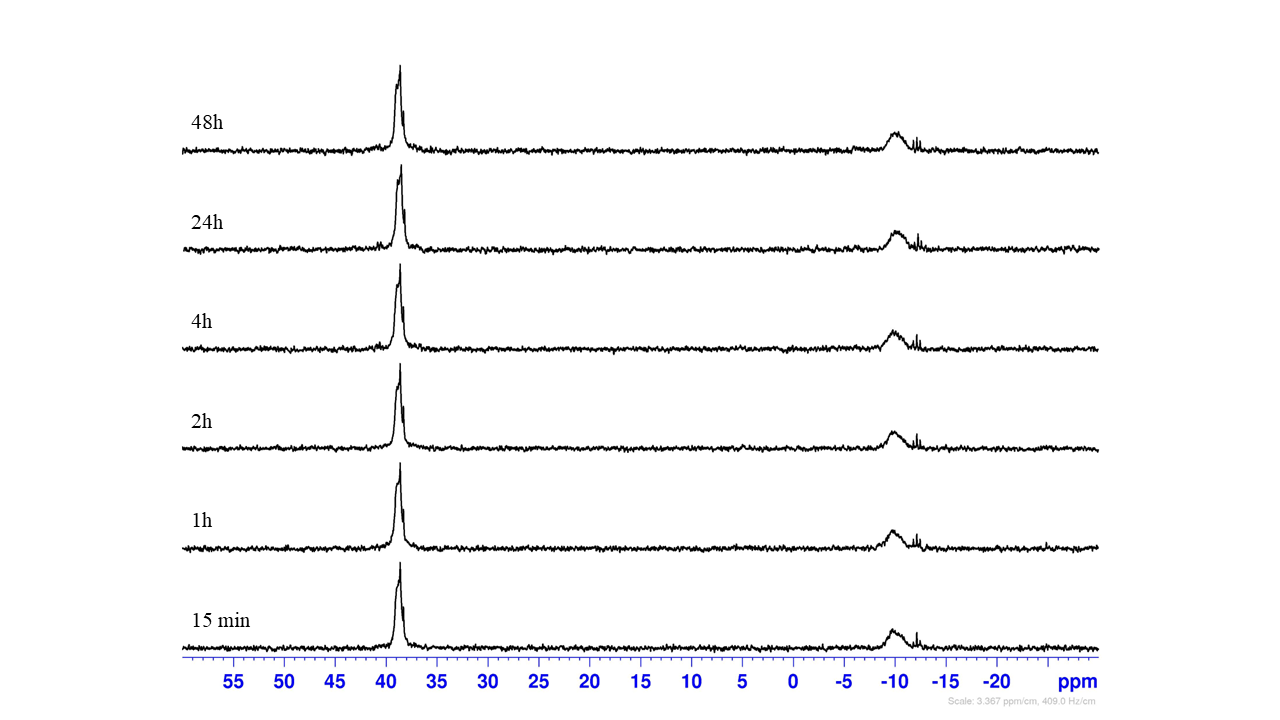


**Figure S20**. ^31^P{^1^H} NMR (121.49 MHz, DMSO-d_6_/D_2_O, 37ºC) of **11** vs. time

**Cell lines and culture conditions**

The MDA-MB-231 and MCF-7 human breast cancer cells were purchased from ATCC. MDA-MB-231 and MCF-7 cells were grown in Dulbecco’s modified Eagles’s medium (DMEM high glucose) (Capricorn Scientific) at 37ºC in 5% CO_2_ supplemented with 10% fetal bovine serum (Capricorn Scientific). All cells were adherent in monolayers and, upon confluence, were washed with phosphate buffer saline (PBS) and harvested by digestion with Trypsin-EDTA 0.05% (v/v). The cells were suspended and transferred into new, sterile, culture flasks, for maintenance or seeded in sterile test microplates for different assays. All cells were manipulated under aseptic conditions in a flow chamber.

**Compound cytotoxicity evaluated using the MTT assay.**

The cells were adherent in monolayers and, upon confluency, were harvested by digestion with trypsin. The cytotoxicity of the complexes against the tumour cells was assessed using the colorimetric assay MTT (3-(4,5-2-yl)-2,5-ditetrazolium bromide), which evaluate the metabolic activity of viable cells. This assay measures the conversion of the yellow tetrazolium into purple formazan by an active mitochondrial dehydrogenase in living cells. For this purpose, cells (10-20 $\times$ 10^3^ in 200 μL of medium) were seeded into 96-well plates and incubated in a 5% CO_2_ incubator at 37ºC. The cells were settled for 24 h followed by the addition of a dilution series of the complexes in medium (200 μL). The complexes were first solubilized in 100% DMSO, given a 10 mM stock solution, and then in medium within the concentration range of 0.1–100 µM. DMSO did not exceed 1% even for the higher concentration used and was without cytotoxic effects. After 24 h of incubation, the treatment solutions were removed by aspiration, and MTT solution (200 μL, 0.5 mg·mL^-1^ in PBS) was added to each well. After 3 h at 37ºC/5% CO_2_, the solution was removed, and the purple formazan crystals formed inside the cells were dissolved in DMSO (200 μL) by thorough shaking. The cellular viability was evaluated by measuring the absorbance at 570 nm by using a microplate spectrophotometer. The IC_50_ values were obtained by dose-response curves using the GraphPad Prism software (vs.5).

**Cell death measurement using flow cytometry – the annexin V/PI assay.**

After a 24 h treatment with compounds **9**, **10** and **11**, both suspended and attached cells were collected and washed with PBS. The cells were resuspended in 200 μL of 1$\times$ binding buffer and were incubated with 5 μL of FITC annexin V (BD Biosciences, San Jose, CA, USA) and 10 μL of PI (50 mg·mL^-1^) for 20 min in the dark. The samples were analysed by using fluorescence-activated cell sorting (FACS) using a Beckman Coulter EPICS XL-MCL. All data were analysed using the FlowJo software (version 10, Tree Star Inc.).

**Complex uptake and distribution by ICP-MS.**

For the cellular uptake experiments, MDA-MB-231 cells (approx. 5·10^6^ cells / 5 mL medium) were exposed to the complexes at their respective IC_50_ concentrations and at two conditions (6 h and 24 h of incubation time)., then washed with ice-cold PBS and centrifuged to obtain a cellular pellet.[7] The cytosol, membrane/particulate, cytoskeletal and nuclear fractions were extracted using a FractionPREP™, cell fractionation system (BioVision, USA) and performed according to the manufacturer's protocol. The Ru (^101^Ru) and Pd (^106^Pd) contents in the different fractions were measured by a Thermo X-Series Quadrupole ICP-MS (Thermo Scientific) after digestion of the samples. Briefly, samples were digested with ultrapure HNO_3_ (65%), H_2_O_2_ and H_3_PO_4_ in a closed pressurized microwave digestion unit (Mars5, CEM) with medium pressure HP500 vessels and then diluted in ultrapure water to obtain 2.0% (v/v) nitric acid. The instrument was tuned using a multielement ICP-MS 71C standard solution (Inorganic Venture). Indium (115In) at 10μML^−1^ was used as internal standard.

**Binding interaction with Human Serum Albumin by Steady-State and Time-Resolved Fluorescence Emission.**

The stock solutions of HSA were freshly prepared for each experiment by gently dissolving the protein in PBS pH 7.4 (tablets from Fisher) for 1 hour to allow the protein to completely hydrate. The concentration of protein in the stock solutions was determined by spectrophotometry using the molar absorption coefficient at 280 nm ε_280_(HSA) = 36,850 M^−1^ cm^−1^.[8]

In each essay all spectroscopic measurements were carried out on individually prepared samples to ensure the same pre-incubation time at (37.0 ± 0.1) °C, the same % of DMSO in the final samples, the same exposure to excitation light and to avoid the need for dilution corrections. Moreover, for a direct titration protocol, a highly concentrated stock solution of the compounds in buffer would be required, which would raise solubility issues. Dimethylsulfoxide (DMSO, from Fisher) was used to prepare concentrated stock solutions of each complex, followed by appropriate dilution to obtain the desired concentration. The DMSO content was kept at 2% (*v/v*) in PBS pH 7.4 in all samples. Dilutions were carried out immediately before sample preparation.


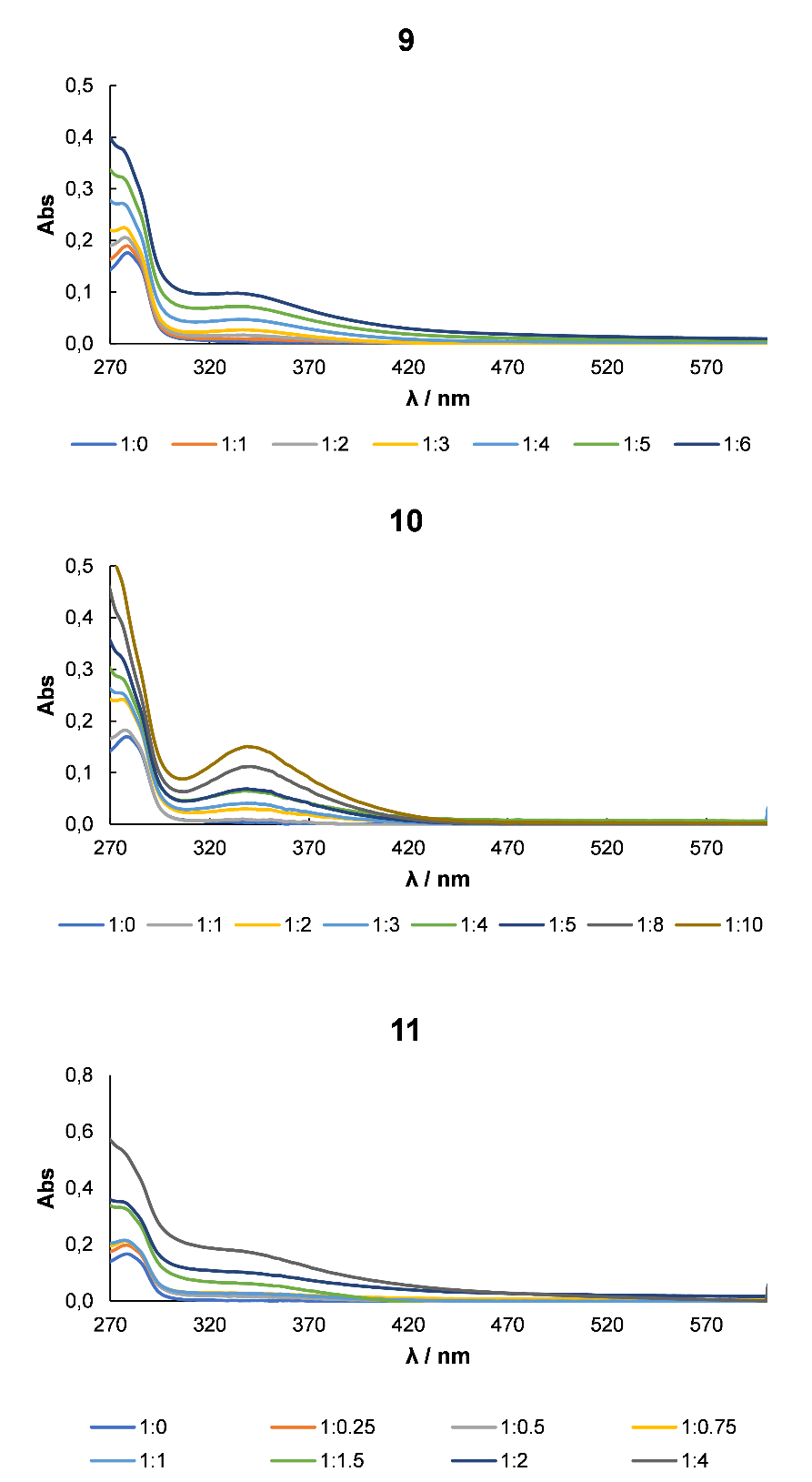


**Figure S21**. Absorbance spectra of HSA in the presence of increasing concentrations of **9**, **10** and **11** after an incubation of 24 h at 37°C. [Conditions: PBS pH 7.4/2% (v/v) DMSO; C_HSA_ = 3.72 µM for **9**, and 5.0 µM for **10** and **11**; λ_exc_ = 295 nm; spectra recorded at (25.0 ± 0.1) °C.

Fluorescence measurements were performed at (25.0 ± 0.1) °C. The final protein concentration in the individually prepared samples (3.72 to 5 μM) was kept constant in each assay, and the complex concentration was varied to obtain the desired HSA: Ru-complex molar ratios ranging from 1:0.25 to 1:10. A control sample containing the same amount of protein, final DMSO content and with no complex was also prepared for each experiment. Appropriate blank samples with no protein but with the same complex concentrations were prepared as well for background correction.

The steady-state fluorescence intensity was measured with excitation at 295 nm, and emission spectra collected with bandwidths of 4 nm for both the excitation and emission. For Stern-Volmer plots, the fluorescence intensity with emission at 340 nm was selected because it is near the maximum emission wavelength (so the sensitivity is close to maximum), but it is further to the red in relation to the water Raman scattering peak.[9] These values were corrected for the absorption and emission inner filter effects[10] using the absorbance recorded for each sample at the excitation and emission wavelengths used for the steady-state quenching analysis. For time-resolved fluorescence measurements, the single photon counting technique was used with a nanoLED N-280 (Horiba Jobin Yvon) as the pulsed excitation source (280 nm), and with emission collected at 350 nm (13 nm emission bandwidth), completely eliminating the contribution of the emission by tyrosine residues.


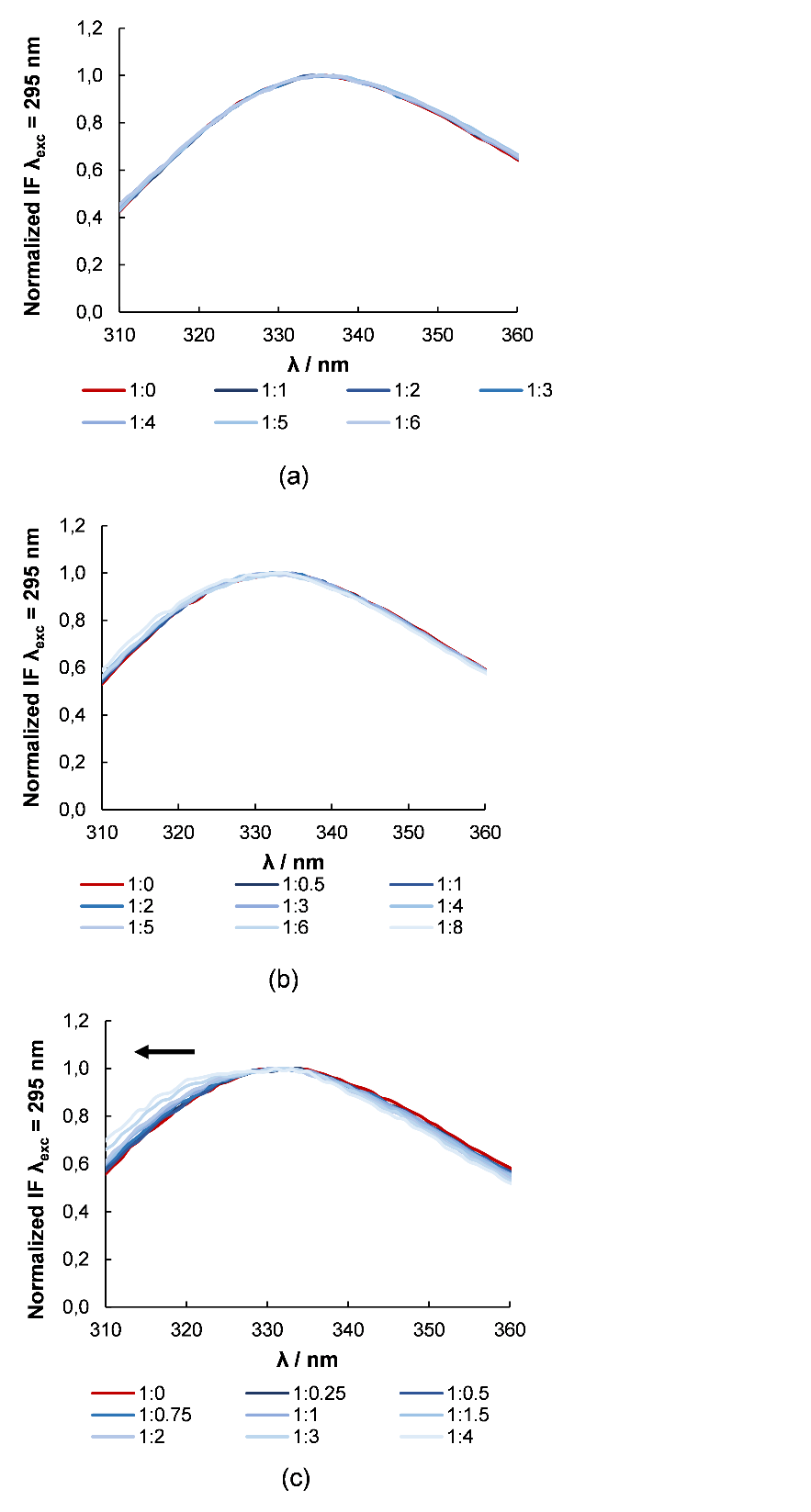


**Figure S22**. Normalized emission spectra of Trp214HSA in the absence (red) and in the presence (blue) of increasing concentrations of **9** (a), **10** (b) and **11** (c) after an incubation of 24 h at 37°C (arrows indicate the change with increasing complex concentration). [Conditions: PBS pH 7.4/2% (v/v) DMSO; C_HSA_ = 3.72 µM for (a), and 5.0 µM for (b) and (c); λ_exc_ = 295 nm; spectra recorded at (25.0 ± 0.1) °C.

The experimental fluorescence intensity decays of HSA in buffer solution can be described by a sum of exponentials:

| $I\left( t \right)= \sum_{i=1}^{n} p_{i}\exp(-\frac{t}{\tau_{i}})$ | (1) |
| --- | --- |

where *p*_i_ and τ_i_ are the pre-exponential factors and lifetime of component *i*, respectively. The amplitude or normalized pre-exponential of each lifetime component *α_i_* is *p_i_*/Σ*_i_* *p_i_* with Σ*_i_* *α_i_* = 1. Fluorescence decays were analyzed by an iterative deconvolution method using the *TRFA Data Processor software* (version 1.4; Minsk, Belarus) and the instrument response function was obtained using the scattering by a colloidal suspension of silica (Ludox^®^, Sigma-Aldrich) diluted in water. Criteria for judging the quality of the fit were a reduced χ^2^ close to 1, and a random distribution of weighted residuals and residuals autocorrelation.

To evaluate changes in the quantum yield by processes affecting the fluorescence lifetimes of HSA, the *amplitude-weighted mean fluorescence lifetime* ($\bar{\tau}$) was calculated using equation (2).

| $\bar{\tau}= \sum_{i=1}^{n} \alpha_{i}\tau_{i}$ | (2) |
| --- | --- |


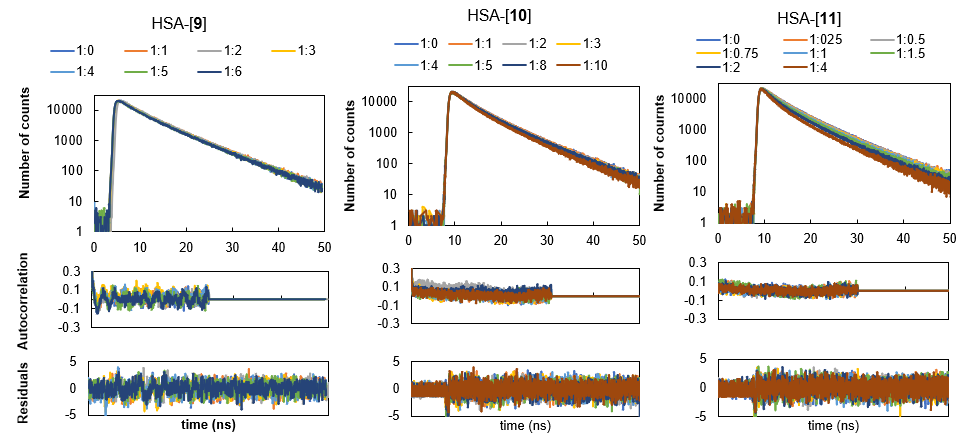


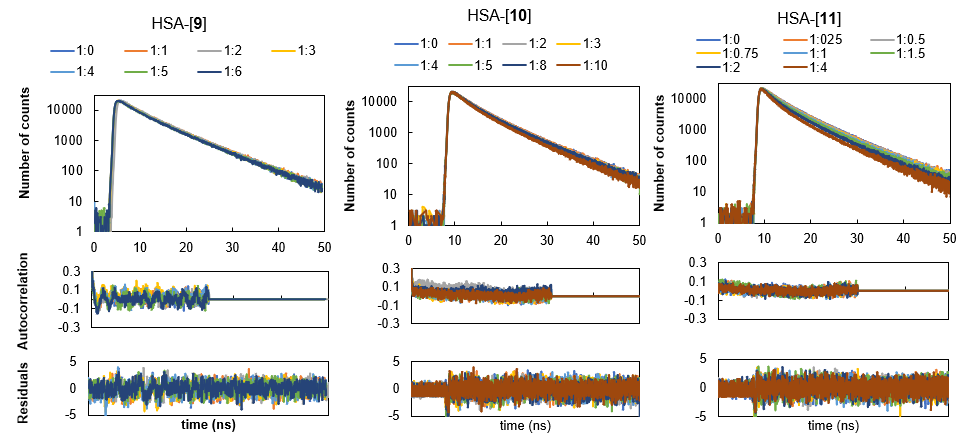


**Figure S23**. Fluorescence intensity decays of Trp214-HSA with compounds **9, 10** and **11.** Top panels: experimental decays. Bottom panels: autocorrelation and residues. Trp214-HSA fluorescence decays were obtained as described in the experimental procedures*.* [Conditions: PBS pH 7.4/2% (v/v) DMSO; C_HSA_ = 3.72 µM for **9**, and 5.0 µM for **10** and **11**; λ_exc_ = 280 nm; λ_em_ = 350 nm decay acquired at (25.0 ± 0.1) °C.]
